# Application of a Fluorescence Anisotropy-Based Assay to Quantify Transglutaminase 2 Activity in Cell Lysates

**DOI:** 10.3390/ijms23094475

**Published:** 2022-04-19

**Authors:** Sandra Hauser, Paul Sommerfeld, Johanna Wodtke, Christoph Hauser, Paul Schlitterlau, Jens Pietzsch, Reik Löser, Markus Pietsch, Robert Wodtke

**Affiliations:** 1 Helmholtz-Zentrum Dresden-Rossendorf, Institute of Radiopharmaceutical Cancer Research, Bautzner Landstraße 400, 01328 Dresden, Germany; s.hauser@hzdr.de (S.H.); j.wodtke@hzdr.de (J.W.); paul.schlitterlau@aol.de (P.S.); j.pietzsch@hzdr.de (J.P.); r.loeser@hzdr.de (R.L.); 2 Institute II of Pharmacology, Center of Pharmacology, Faculty of Medicine and University Hospital of Cologne, University of Cologne, Gleueler Straße 24, 50931 Cologne, Germany; paul.sommerfeld@uk-koeln.de (P.S.); christoph.hauser1@gmx.de (C.H.); 3 Faculty of Chemistry and Food Chemistry, School of Science, Technische University Dresden, Mommsenstraße 4, 01069 Dresden, Germany

**Keywords:** activity-based protein profiling, cancer, ELISA, enzyme assay, transamidase activity

## Abstract

Transglutaminase 2 (TGase 2) is a multifunctional protein which is involved in various physiological and pathophysiological processes. The latter also include its participation in the development and progression of malignant neoplasms, which are often accompanied by increased protein synthesis. In addition to the elucidation of the molecular functions of TGase 2 in tumor cells, knowledge of its concentration that is available for targeting by theranostic agents is a valuable information. Herein, we describe the application of a recently developed fluorescence anisotropy (FA)-based assay for the quantitative expression profiling of TGase 2 by means of transamidase-active enzyme in cell lysates. This assay is based on the incorporation of rhodamine B-isonipecotyl-cadaverine (**R-I-Cad**) into *N*,*N*-dimethylated casein (DMC), which results in an increase in the FA signal over time. It was shown that this reaction is not only catalyzed by TGase 2 but also by TGases 1, 3, and 6 and factor XIIIa using recombinant proteins. Therefore, control measurements in the presence of a selective irreversible TGase 2 inhibitor were mandatory to ascertain the specific contribution of TGase 2 to the overall FA rate. To validate the assay regarding the quality of quantification, spike/recovery and linearity of dilution experiments were performed. A total of 25 cancer and 5 noncancer cell lines were characterized with this assay method in terms of their activatable TGase 2 concentration (fmol/µg protein lysate) and the results were compared to protein synthesis data obtained by Western blotting. Moreover, complementary protein quantification methods using a biotinylated irreversible TGase 2 inhibitor as an activity-based probe and a commercially available ELISA were applied to selected cell lines to further validate the results obtained by the FA-based assay. Overall, the present study demonstrates that the FA-based assay using the substrate pair **R-I-Cad** and DMC represents a facile, homogenous and continuous method for quantifying TGase 2 activity in cell lysates.

## 1. Introduction

Activity-based assay methods are fundamental tools for the functional characterization of enzymes [1]. In addition to studying isolated enzymes, the functional quantification of enzymes in biological samples is essential for target discovery and validation as well as drug development, in particular when considering the potential differences in the proteome and functional expression of enzymes [2,3,4,5,6]. An established methodology for determining enzyme activities in cells or homogenates derived thereof is activity-based protein profiling (ABPP) which utilizes covalent probes connected with a reporter tag for signal readout with a desired analytical platform (e.g., in-gel fluorescence scanning after gel electrophoresis using a fluorophore-labeled covalent probe) [7,8,9,10]. Usually, the ABPP probes are designed to target a subset of enzymes, e.g., fluorophosphonates for serine hydrolases, which allows the activity screening of several enzymes in parallel to provide “a global view of the functional state of the proteome” [8].

When focusing on the quantification of one particular enzyme in biological samples, the application of substrate-based activity assays appears more convenient than covalent probes as they allow for a continuous readout accompanied by a higher sensitivity. The prerequisite is, however, that the substrate conversion is specific. The enzyme family of transglutaminases (EC 2.3.2.13) belongs to the class of acyl transferases that catalyze the Ca^2+^-dependent acyl transfer of protein-bound glutamine residues to a variety of primary amines. This enzyme activity is also referred to as transamidase activity. A wide range of primary amines were recognized as acyl acceptors, including protein-bound lysine residues, biogenic amines and polyamines [11,12,13]. Owing to this unique substrate profile, the specific detection of transglutaminases even in complex biological samples becomes possible. In this context, Heinrich Waelsch and colleagues originally discovered this enzyme activity in soluble protein fractions of guinea pig liver by studying the incorporation of ^14^C-labeled amines into endogenous proteins [14,15]. Since then, a variety of activity assays derived from this original method have been developed for measuring transamidase activity in different specimens with a primary focus on the detection of one isozyme in this family: transglutaminase 2 (TGase 2) [16].

TGase 2 receives special attention within the family of transglutaminases as it exhibits several further enzymatic and nonenzymatic functions apart from its transamidase activity [17]. For example, it binds guanine nucleotides and can act as an intracellular G-protein, designated as Gα_h_, which presumably contributes to the survival of cells [18,19]. Moreover, TGase 2 has high affinities for fibronectin [20,21] and integrins [22] and thus, a role in enhancing the interaction of both by acting as a scaffold protein has been proposed [23]. According to its multifunctional character, it appears comprehensible that a complex network of factors exists that regulates the functions of TGase 2. The most striking aspect of this regulation might be the fact that the eponymous function is actually latent under normal conditions, as intracellularly the low Ca^2+^ and high GTP levels suppress the transamidase activity [24,25], while extracellular TGase 2 is oxidized and thus transamidase inactive even though there is a high Ca^2+^ concentration [26,27]. However, this tight regulation is partly lost in physiological stress situations, such as wound healing [26], and different pathophysiological situations, including fibrosis [28,29,30,31] and celiac disease [32,33] in which an aberrant transamidase activity of TGase 2 contributes to the onset and progression of the diseases. Furthermore, transamidase activity has been linked to the survival mechanisms of cancer cells [34,35,36,37]. Apart from its distinct molecular function, increased expression of TGase 2 is often a characteristic of malignant neoplasms and is associated with a poor prognosis [34,38,39,40]. This renders TGase 2 an interesting target for the diagnosis and therapy of such pathophysiological conditions with the major focus being put on transamidase activity-directed compounds for these purposes [16,30,33,41,42].

While in situ transamidase activity in unstimulated cells can usually not be assessed due to the aforementioned regulation mechanisms, the determination of this enzyme activity in cells after targeted activation or in cell/tissue homogenates under favorable conditions for transamidase activity (high Ca^2+^ concentration in the presence of reducing agents) is often a subject of interest. A frequently employed assay principle to measure transamidase activity is based on the incorporation of various amines into casein or *N*,*N*-dimethylated casein (DMC). The amines labeled with an appropriate reporter group for signal readout include radiolabeled polyamines such as ^14^C- or ^3^H-labeled putrescine [43,44,45,46], but also nonradioactive probes such as biotinyl-cadaverine [47,48,49], dansyl-cadaverine [50,51] or Boc-K-NH(CH_2_)_2_NH-dansyl (KXD) [52]. However, these methods are either discontinuous in nature (stopped assays), as in the case of the radiolabeled putrescines and biotinyl-cadaverine, or the signal readout is prone to interferences, as in the cases of dansyl-cadaverine and KXD (e.g., background fluorescence).

**Figure 1:**
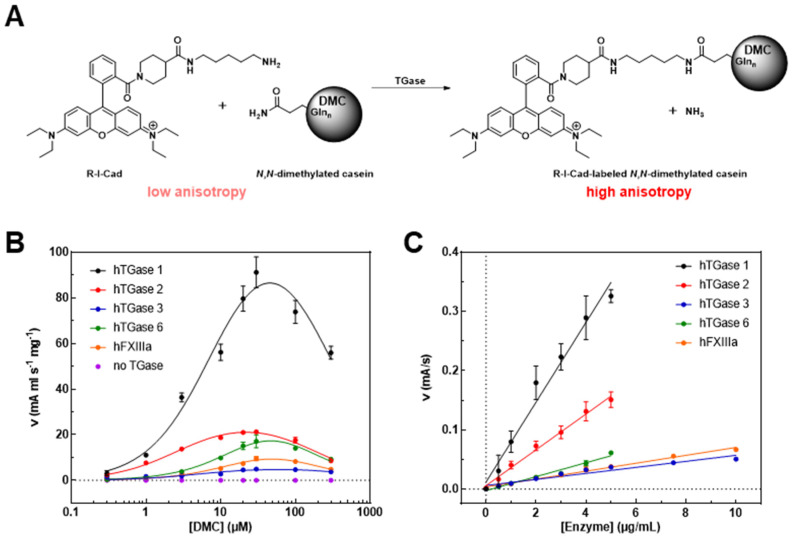
Incorporation of **R-I-Cad** into DMC by different TGases. (**A**) Principle of the FA-based TGase activity assay. (**B**) Plots of rate versus DMC concentration for the reaction of DMC with **R-I-Cad** (0.74 µM) in the absence and presence of different human (h) TGases (2 µg/mL for hTGase 1; 5 µg/mL for hTGases 2, 3, 6 and hFXIIIa). Nonlinear regressions were conducted according to Equation (2) (substrate inhibition; Section 4.3.1). (**C**) Plots of rate versus hTGase concentration for the reaction of DMC (30 µM) with **R-I-Cad** (0.74 µM) with linear regressions. Data shown are mean values ± SEM of 2–3 separate experiments, each performed in duplicate. Conditions: pH 8.0, 30 °C, 5% DMSO, and 500 µM DTT. Data for hTGase 2 in (**B**) and (**C**) have previously been published (Adapted with permission from Wodtke et al. [54]. Copyright 2022 American Chemical Society).

Herein, we demonstrate that our recently developed fluorescence anisotropy (FA)-based assay for purified TGase 2, which uses DMC and rhodamine B-isonipecotyl-cadaverine (**R-I-Cad**, Figure 1A) [53], can be applied to reliably quantify the transamidase activity of TGase 2 in cell lysates. For this purpose, the specificity and accuracy of the FA-based assay for TGase 2 quantification was assessed and a set of 30 cancer and noncancer cell lines were screened in terms of a comparative ABPP. Furthermore, the calculated concentration data (fmol/µg) for selected cell lines were compared to data obtained with an activity-based ELISA using a biotinylated covalent probe, to protein expression data from Western blot analysis, and to data from a commercially available sandwich ELISA.

## 2. Results

### 2.1. Characterization of DMC and R-I-Cad toward Different Transglutaminases

Our previous studies showed that the substrate pair DMC and **R-I-Cad** are suitable to measure the activity of guinea pig (gp) TGase 2 [53] and human (h) TGase 2 [54]. However, for the application of this assay to measure the transamidase activity of TGase 2 in cell lysates, the contribution of other TGases to the overall increase in the FA signal must be considered. Therefore, the substrate properties of DMC and **R-I-Cad** toward other active TGases were assessed (Figure 1B and Table 1). For all studied TGases, i.e., hTGase 1, hTGase 3, hTGase 6 and hFXIIIa, an increase in the FA signal over time was measured. Furthermore, the kinetic characterization of DMC between 0.3 and 300 µM at a fixed concentration of **R-I-Cad** (0.74 µM) revealed an apparent substrate inhibition behavior for all TGases with *K*_i_ values > 100 µM. This was also previously observed for hTGase 2 and gpTGase 2 [53,54]. The *K*_m_ values of DMC range from 2.78 to 17.5 µM and the order from lowest to highest is hTGase 2 < hTGase 3 ≈ hTGase 1 < hTGase 6 ≈ hFXIIIa. For hTGase 1, the higher *K*_m_ value compared to hTGase 2 is compensated by a higher V_max_ value, resulting in similar V_max_/*K*_m_ values, as a measure of the substrate efficiency, for both isoforms. For the other TGases, the V_max_ value is similar (hTGase 6) or lower (hTGase 3 and hFXIIIa) compared to the value for hTGase 2. Consequently, the substrate efficiency of DMC for these TGases is significantly lower (by factors of 6–10).

The difference in the rates of **R-I-Cad** incorporation into DMC can also be derived from measurements at varying enzyme concentrations, but fixed concentrations of DMC (30 µM) and **R-I-Cad** (0.74 µM), as shown in Figure 1C. It is worth noting that a linear dependence of enzymatic activity from TGase concentration exists up to 5 µg/mL for hTGases 1, 2, and 6 and up to 10 µg/mL for hTGase 3 and hFXIIIa.

**Figure 2:**
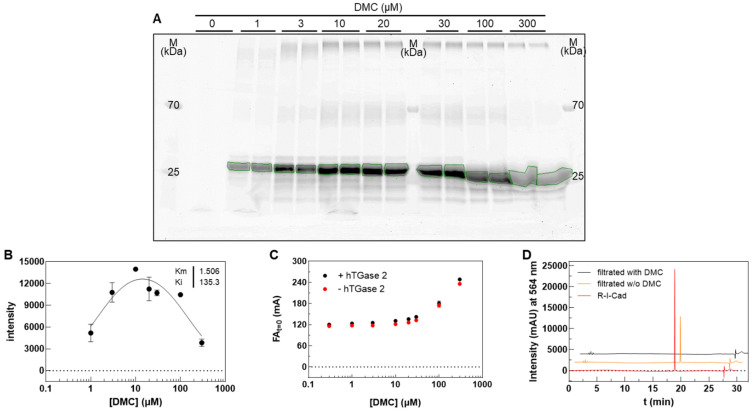
Elucidation of the underlying mechanism for the decreased product formation at high DMC concentrations. (**A**) Visualization of **R-I-Cad**-labeled DMC at varying DMC concentrations after separation of the reaction mixtures (one gel, each DMC concentration in duplicate, conditions as described in Figure 1 but with 2.5 µg/mL hTGase 2) with SDS-PAGE and detection by in-gel fluorescence readout (Cy3 filter of Amersham Typhoon 5 biomolecular imager). Regions bordered in green were quantified for the plot in (**B**). The 25 and 70 kDa orange-stained proteins of the Thermo Scientific™ PageRuler™ Plus Prestained Protein Ladder were made visible by the fluorescence readout. (**B**) Plot of Intensity (DMC bands in (**A**), mean values ± SD) versus DMC concentration with nonlinear regression using Equation (2). (**C**) Plot of the FA values (mean values ± SEM of two separate experiments, each performed in duplicate) at start of measurement versus DMC concentration. The data were obtained during the characterization of DMC on hTGase 2 previously published by us [54]. (**D**) Analytical RP-HPLC chromatograms at 564 nm for a solution of **R-I-Cad** in MOPS buffer (8.1 µM, 5 µL analyzed, red) and after filtration (15,000× *g*, 90 min) of this solution using centrifugal concentrators (polyethersulfone membrane, molecular weight cut-off of 3 kDa) in the absence (20 µL analyzed, orange) and presence (20 µL analyzed, black) of DMC (300 µM).

The apparent substrate inhibition behavior at high DMC concentrations using **R-I-Cad** as an amine donor was also observed for the related **R-S-Cad** (sarcosine linker between cadaverine and rhodamine B) but not for **F-Cad** (FITC-labeled cadaverine) toward hTGase 2 [53]. This raises the question of whether the enzymatic reaction rate is really diminished at high concentrations of DMC in the presence of the rhodamine B-labeled cadaverines or if another phenomenon causes the observed kinetic behavior. To address this question, we examined the hTGase 2-catalyzed formation of **R-I-Cad**-labeled DMC by separating the reaction mixtures after the time span of the FA measurements using SDS-PAGE and detection of the reaction product by in-gel fluorescence measurement (Figure 2A). The quantification of the detected fluorescent bands and plotting the obtained intensities versus the DMC concentration revealed a similar curve pattern and comparable *K*_m_ and *K*_i_ values as obtained for the FA readout (Figure 2B). Therefore, the rate of product formation at high DMC concentrations is indeed reduced, which actually points to the phenomenon of substrate inhibition. However, we also noticed for **R-I-Cad** that the FA values at start of measurement steadily increased with increasing DMC concentration (Figure 2C). This phenomenon is also seen for **R-S-Cad** and **F-Cad**, but it is significantly less pronounced for the fluorescein conjugate compared to the rhodamine B conjugates (Figure S1 in Supplementary Materials). Moreover, this increase does not result from a delayed start of the measurements (<10 s) as the initial FA values are also increased in the absence of TGase 2 (Figure 2C). The data rather suggest that **R-I-Cad** (and also **R-S-Cad**) binds noncovalently to DMC to a large extent at high concentrations of the protein which in turn results in an increase in the FA signal. To prove the high extent of noncovalent binding of **R-I-Cad** to DMC, an ultrafiltration experiment of dissolved **R-I-Cad** (8.1 µM) in the presence and absence of DMC (300 µM) was performed using centrifugal concentrators with a semipermeable membrane (polyethersulfone, molecular weight cut-off of 3 kDa) and the eluates were analyzed with RP-HPLC (Figure 2D). Due to the high binding of **R-I-Cad** to the filter membrane, a ~10-times higher concentration of **R-I-Cad** compared to the FA-based assay was used. As shown in Figure 2D, **R-I-Cad** was not detectable in the eluate when filtrated in the presence of DMC. This result, together with the increased FA values, indicate that the decreased rate of product formation at high DMC concentrations originate from a reduction in the free **R-I-Cad** concentration, which in turn leads to substrate depletion. Considering the previously measured dependence of the FA rate from the concentration of **R-I-Cad** (at 30 µM DMC) [53], which revealed a constant FA rate from 0.0024 to 0.81 µM, the concentration of free **R-I-Cad** at 300 µM DMC seemed to drop by a factor greater than 300.

### 2.2. Quantification of TGase 2 Activity in Cell Lysates by the FA-Based Assay

**Figure 3:**
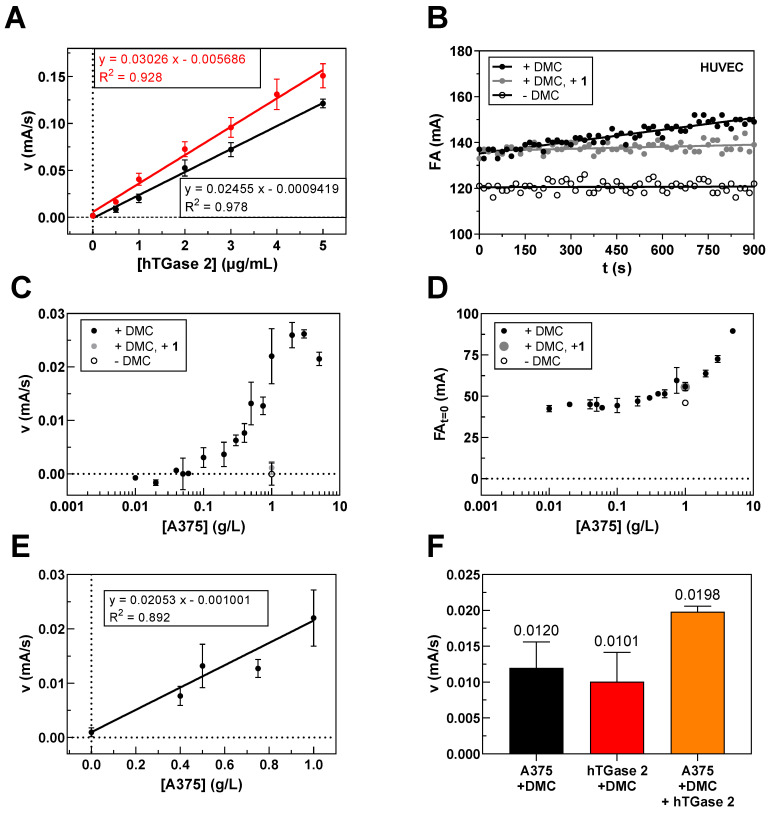
Assessing the accuracy of the FA-based assay to determine the TGase 2 activity in cell lysates. (**A**) Plot of rate versus concentration of recombinant hTGase 2 in MOPS buffer (red) and in a 1:1 mixture of modified RIPA and MOPS buffers (black) with linear regression to the data. Data shown are mean values ± SEM of two separate experiments, each performed in duplicate. Slopes are not significantly different (unpaired *t* test, *p* = 0.189 two-tailed). (**B**) Exemplary plot of FA over time for the reaction of **R-I-Cad** in the presence of HUVEC lysate (0.5 g protein/l) with linear regression to the data to obtain rate values for the FA increase (v in mA/s). To prove that the FA increase over time mainly originates from the activity of TGase 2, control measurements in the presence of the selective TGase 2 inhibitor **1** were performed. Moreover, the necessity of adding DMC as acyl donor to achieve a measurable FA increase was shown by control measurements in the absence of DMC. Data shown are single measurements. (**C**) Plot of rate versus concentration of A375 lysate (0–5 g/L). Data shown are mean values ± SD of 2–6 measurements. (**D**) Plot of the FA values at start of measurement versus concentration of A375 lysate for the same data set as in (**B**). (**E**) Plot of rate versus concentration A375 lysate (0, 0.4–1.0 g/L) with linear regression to the data. (**F**) Results of a “spike and recovery” experiment. hTGase 2 (0.5 µg/mL) was spiked to A375 lysate (0.5 g/L, orange) and the observed rate in FA increase is compared to the rates observed for A375 lysate (black) and hTGase 2 (red) alone. Data shown are mean values ± SD of 2–6 measurements. Conditions: pH 8.0, 30 °C, 2.7 mM CaCl_2_, 5% DMSO, 500 µM DTT (cell lysates) or TCEP (recombinant hTGase 2), 30 µM DMC, 0.81 µM **R-I-Cad**, and 10 µM **1** (preincubation period: 10 min).

For preparation of the cell lysates, cell pellets were initially resuspended in standard radioimmunoprecipitation assay buffer (RIPA lysis buffer: 150 mM NaCl, 50 mM Tris pH 8.0, 1% NP40, 0.5% SDS (*w/v*), 0.5% sodium deoxycholate (*w/v*), 1 µg/mL leupeptin, 1 mM PMSF, 1 mM Na_3_VO_4_, 1 mM DTT, and 5 mM NaF) incubated on ice for 30 min and applied to ultrasound. However, control measurements with recombinant hTGase 2 in a 1:1 mixture of RIPA and MOPS buffer (100 mM MOPS, pH 8.0, 6 mM CaCl_2_) showed no measurable FA increase over time in contrast to MOPS buffer alone (data not shown). We hypothesized that the present detergents, NP40, SDS, and sodium deoxycholate, might prevent TGase 2 activity. Indeed, the omission of these additives restored the TGase 2 activity to a similar level as found for MOPS buffer alone (Figure 3A) and the modified RIPA buffer was used for cell lysis. From the progress curves of FA increase over time at varying concentrations of hTGase 2, the limit of detection for measurements up to 900 s was defined to be 0.5 µg/mL hTGase 2 as the corresponding FA rate (0.008 ± 0.005 mA/s) is roughly four times that of the background (0.002 ± 0.002 mA/s).

**Figure 4:**
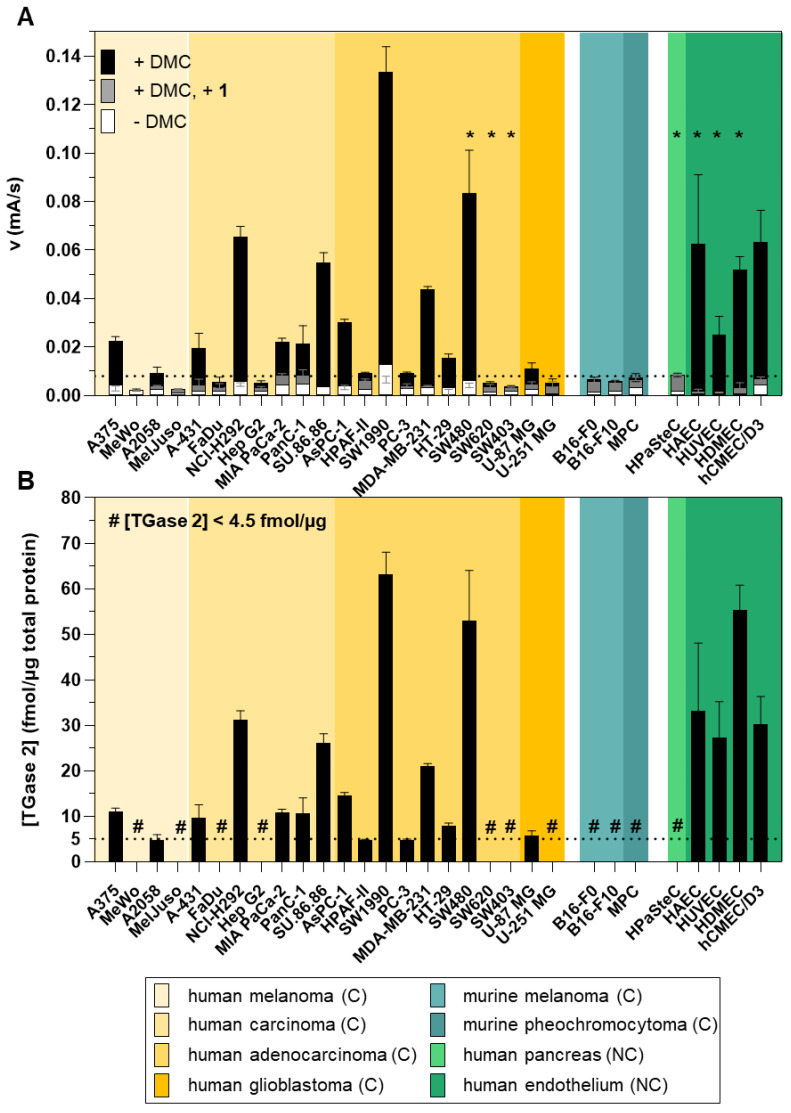
TGase activity determined by FA-based assay in lysates of different human and mouse cancer cell lines (C) and human noncancer cell lines (NC). (**A**) Summary of FA rates (mean values ± SEM of 2–5 separate experiments, each performed in duplicate) for the reaction of DMC (30 µM) with **R-I-Cad** (0.81 µM) in the presence of different cell lysates (2 g/L). For some cell lines (marked with *), lysate concentrations of 0.5 g/L (HDMEC and HUVEC), 1 g/L (HAEC and HPaSteC) or 1.5 g/L (SW480, SW620, and SW403) were used. The dotted line indicates an FA rate value of 0.008 mA/s, which is the detection limit to reliably detect an increase in the FA signal over time. (**B**) Summary of calculated hTGase 2 concentrations (mean values in fmol/µg total protein ±SEM) in the different cell lines based on the FA rate values “+DMC” (black bars in (**A**). For cell lines marked with #, the FA rate lies below the detection limit and thus only an upper limit for the respective TGase 2 concentration can be given. For (**A**,**B**), cell lines were sorted by tumor type and indicated by different colors as shown in the legend. Conditions: pH 8.0, 30 °C, 2.7 mM CaCl_2_, 5% DMSO, 500 µM DTT, 30 µM DMC, 0.81 µM **R-I-Cad**, 10 µM **1** (preincubation period: 10 min).

After having established the buffer conditions for the measurements in cell lysates, we proceeded with the screening of various cell lines by the FA-based assay. An exemplary progress curve for the FA increase over time using DMC (30 µM) and **R-I-Cad** (0.81 µM) in the presence of HUVEC lysate (0.5 g/L) is shown in Figure 3B. The recognition of DMC and **R-I-Cad** as substrates by various TGases (see Section 2.1) required control measurements in the presence of a selective inhibitor of hTGase 2 to assess the contribution of TGase 2 to the overall signal. For this purpose, we chose the irreversible inhibitor **1** as it exhibits excellent selectivity for hTGase 2 [55], which is also confirmed herein (see Section 2.3). A further control experiment was included, in which DMC was omitted to investigate the necessity of adding DMC as acyl donor. It is worth noting that both the presence of **1** (10 µM) and the absence of DMC reduced the observed FA increase over time below the detection limit for all studied cell lines (Figure 3B and Figure 4A).

We envisaged to transform the obtained FA rates into more familiar concentration values of TGase 2 (fmol/µg protein lysate) using the standard curve shown in Figure 3A. For this purpose, it was necessary to assess the accuracy of the FA-based assay for determining the TGase 2 activity in a quantitative manner (Figure 3C–F). These experiments were exemplarily conducted with lysates derived from A375 cells. Firstly, the dependence of the FA rate from the A375 lysate concentration (0.01–5 g/L) was investigated. As shown in Figure 3C, the FA rate increased with increasing lysate concentration but reached a plateau at 2 g/L and even declined at 5 g/L. Moreover, the FA values at start of measurement increased with increasing concentration of the cell lysate (Figure 3D). Similar curve patterns were obtained for the dependence of the FA rate and the initial FA values from the DMC concentration (Section 2.1) and can be attributed here to noncovalent binding of **R-I-Cad** to cellular proteins, which becomes relevant at high protein concentrations and might result in a reduction in the enzymatic rate due to substrate depletion by nonspecific protein binding. From the obtained relationship between the concentration of A375 lysate and FA rate shown in Figure 3B, it can be concluded that for most of the cell lysate measurements the observed FA rate underestimates the true TGase 2 concentration as usually a lysate concentration of 2 g/L was applied. To estimate this factor, the FA rates for A375 lysate concentrations from 0.4 g/L (FA rate above detection limit) up to 1 g/L were plotted against the respective lysate concentrations (Figure 3E), which yielded a rather good linear relationship. By extrapolating this relationship up to 2 g/L of A375 cell lysate, an FA rate of 0.040 mA/s was calculated. The measured mean value for A375 cell lysate (Figure 4A) was 0.022 mA/s. Therefore, the actual TGase 2 activity might be higher by a factor of 1.8. This factor was then applied to the FA rates which were measured at lysate concentrations of 2 g/L to calculate the TGase 2 concentration in the cell lysates (Figure 4B). The same factor was applied to the three cell lines, which were measured at 1.5 g/L lysate concentration (SW480, SW620, and SW403), even though the FA rate at 1.5 g/L of A375 cell lysate was not measured, as the maximal FA rate might be already reached at this concentration (Figure 3C).

A “spike and recovery” experiment was performed to assess that at lysate concentrations ≤ 1 g/L the detection of the TGase 2 activity is not affected by the lysate matrix compared to conditions for the standard curve (Figure 3F). For this purpose, a defined concentration of recombinant hTGase 2 (0.5 µg/mL) was spiked to A375 cell lysate (0.5 g/L) and the obtained FA rate (0.020 mA/s) was compared to the FA rates obtained for A375 cell lysate (0.012 mA/s) and recombinant hTGase 2 (0.010 mA/s) alone, which clearly showed that the FA rates behave proportionally.

A total of 30 cell lines were screened for their TGase 2 activity in the respective lysates (0.5–2.0 g/L) by the FA-based assay (Figure 4A). The cell lines consisted of 22 human cancer cell lines (highlighted in yellow), 3 mouse cancer cell lines (highlighted in cyan) and 5 human noncancer cell lines (highlighted in green). For 11 cell lines (MeWo, MelJuso, FaDu, Hep G2, SW620, SW403, U-251 MG, B16-F0, B16-F10, MPC, and HPaSteC), the observed overall and/or TGase 2-mediated FA increase was below the detection limit. The highest TGase 2 activity was measured in lysates from SW1990 followed by SW480, while the lowest measurable TGase 2 activity was obtained in HT-29 and U87 lysates. After having proved that the transformation of the FA rates into TGase 2 concentrations is possible, this was carried out for all cell lines with an FA rate above the detection limit (Figure 4B). For the SW1990 lysate, which had the highest FA rate, a TGase 2 concentration of 63 fmol/µg was derived. Slightly lower values were obtained for HDMEC (55 fmol/µg) and SW480 (53 fmol/µg) lysates. NCI-H292, SU.86.86, MDA-MB-231, HAEC, HUVEC, and hCMEC/D3 lysates exhibited TGase 2 concentrations between 21 and 33 fmol/µg, while the lowest concentrations were derived for A375, A2058, A-431, MIA PaCa-2, PanC-1, As-PC1, HPAF-II, PC3, HT-29, and U-87 MG, with values between 4.5 and 15 fmol/µg. 

### 2.3. Quantification of TGase 2 Activity in Cell Lysates by an Activity-Based ELISA

**Figure 5:**
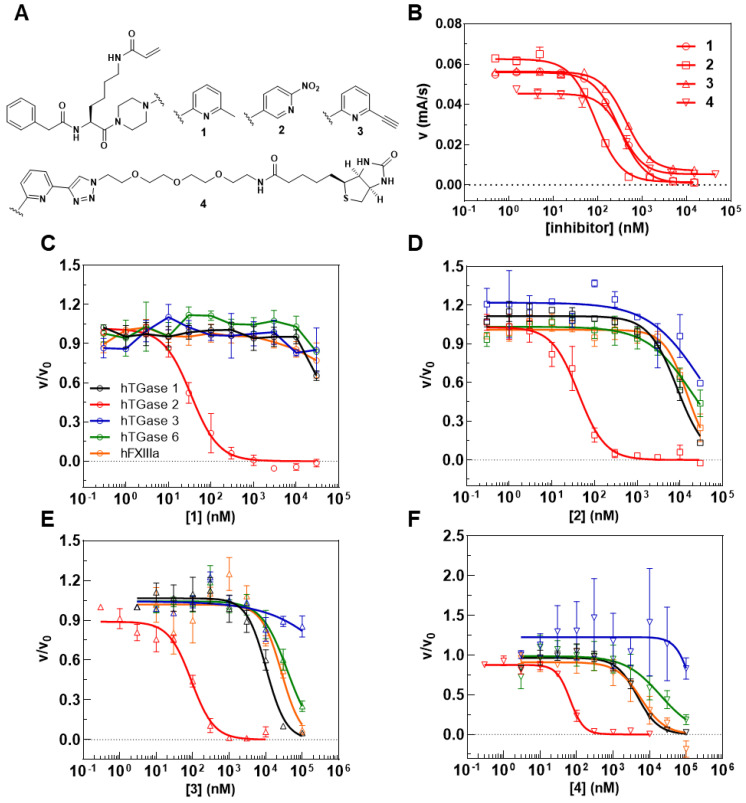
Structures of the inhibitors **1–4** (**A**) and their inhibitory potentials towards different transglutaminases (**B**–**F**). (**B**) Plots of rate versus inhibitor concentration with nonlinear regression using equation III for the reaction of DMC (30 µM) with **R-I-Cad** (0.81 µM) in the presence of hTGase 2 (2 µg/mL) using a preincubation period of 5 min for hTGase 2 and inhibitor. Plots of relative rate versus inhibitor concentration for **1** (**C**), **2** (**D**), **3** (**E**), and **4** (**F**) with nonlinear regressions using Equation (3) (but connecting data points for **1** toward hTGases 1, 3, 6 and hFXIIIa) for the reaction of DMC (30 µM) with **R-I-Cad** (0.74 µM) in the presence of different hTGases (2 µg/mL for hTGase 1; 5 µg/mL for hTGases 2, 3, 6 and hFXIIIa) using a preincubation period of 30 min for hTGases and inhibitor. Data shown are mean values ± SEM of 3 separate experiments, each performed in duplicate. Conditions: pH 8.0, 30 °C, 5% DMSO, 500 µM DTT or TCEP (hTGase 2). Data for inhibitors **1** and **2** shown in **B** were published previously [54].

To validate the concentration data for TGase 2 obtained by the FA-based assay, we envisaged an activity-based ELISA using a biotinylated irreversible inhibitor. Two aspects for incorporation of biotin were considered: (I) substitution of the methyl group in inhibitor **1** might be most favorable as larger substituents such as *tert*-butyl and phenyl groups are well tolerated in this position [54], and (II) the incorporation should take place as the final synthesis step. Therefore, the alkyne-functionalized inhibitor **3** (Figure 5A) was designed, which allows the late-stage modification with an azide-functionalized biotin building block by copper-catalyzed azide-alkyne cycloaddition (CuAAC). The synthesis of **3** was accomplished according to the previously published synthesis route for *N*^ε^-acryloyllysine piperazides, which exploits *N*^α^-Boc-*N*^ε^-acryloyllysine and the respective pyridylpiperazine as central building blocks (Scheme S1 in Supplementary Materials). In this context, the intermediate 4-Boc-1-(6-ethinylpyridin-2-yl)piperazine (**5**) was obtained by the Sonogashira coupling of the bromo analogue of **5** with trimethylsilylacetylene followed by treatment with tetrabutylammonium fluoride for removal of the trimethylsilyl protecting group. Compound **3** was obtained in good yield of 24% over six synthesis steps. Biotin-PEG3-azide was chosen as biotin building block and coupling with **3** was conducted under common conditions for CuAAC reactions (CuSO_4_ × 5 H_2_O, THPTA, sodium ascorbate, Scheme S1) which furnished compound **4** (Figure 5A) in a yield of 51% after purification by RP-HPLC.

The inhibitory potential of compounds **3** and **4** was investigated by means of the FA-based assay [53,54] and a recently developed fluorimetric activity assay [56]. For the FA-based assay, a preincubation period of 5 min provided IC_50_ values of 441 and 249 nM for compounds **3** and **4**, respectively, which indicates that the biotin conjugate is more potent than the alkyne precursor and even inhibitor **1** (Figure 5B and Table 2). In line with these results, *k*_inact_/*K*_I_ values (performance constants) of 3450 and 7260 M^−1^s^−1^ were obtained for **3** and **4**, respectively (*k*_inact_/*K*_I_ value of **1** is 4880 M^−1^s^−1^; Table 2). For assessing the selectivity of inhibitors **3** and **4**, we took advantage of the suitability of **R-I-Cad** and DMC to act also as substrate pair toward hTGase 1, hTGase 3, hTGase 6 and hXIIIa. The preincubation period was set to 30 min due to the expected lower reactivity toward these TGases. As expected, the IC_50_ values toward hTGase 2 were lower after 30 min preincubation period but the relative difference remained similar (96 nM for **3** and 68 nM for **4**). In contrast, the IC_50_ values toward the other TGases are in the single-digit to triple-digit µM range, resulting in selectivity factors from 106 to 1000 for **3** and 67–1500 for **4** (Figure 5C and Table 2). It is worth noting that the highest reactivity was seen toward hTGase 1 and hFXIIIa, which furthermore was more pronounced for **4** compared to **3**. A similar selectivity profile was obtained for compound **1** and the nitropyridylpiperazine derivative **2** (Figure 5C and Table 2). In this context, inhibitor **2** is one of the most potent inhibitors identified in our previous SAR study [54], but its selectivity profile was not assessed so far.

**Figure 6:**
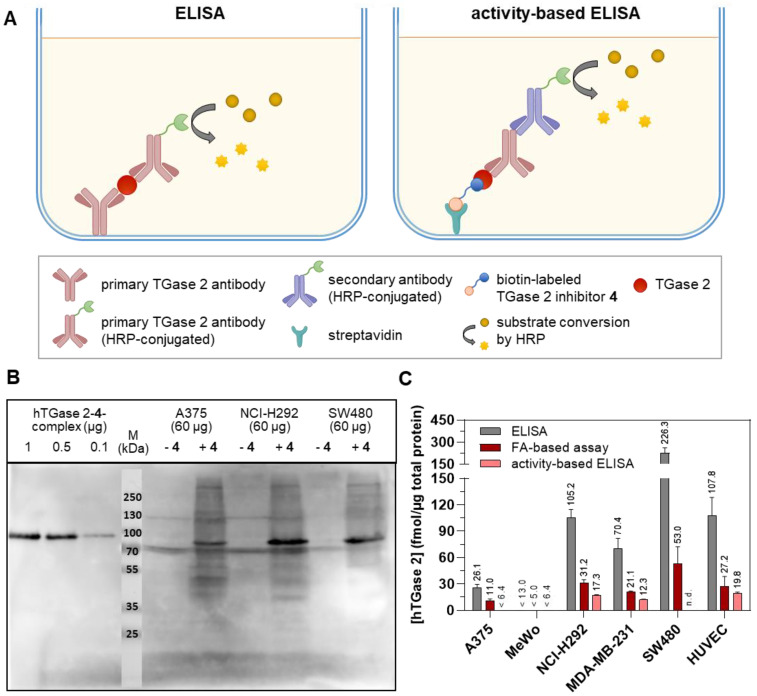
Overview of the applied ELISA methods and hTGase 2 concentrations obtained with these methods. (**A**) Left: Principle of the applied commercially available two-site sandwich ELISA (Zedi*Xclusive* Tissue Transglutaminase EIA, E018) from Zedira^®^ to detect hTGase 2. Right: Principle of the developed activity-based ELISA using biotinylated inhibitor **4** and streptavidin-coated well plates to detect active hTGase 2. The procedures for both methods are described in Section 4.5 and Section 4.7, respectively. (**B**) Visualization of recombinant hTGase 2 and hTGase 2 in cell lysates with inhibitor **4** (20 µM) after separation of the reaction mixtures with SDS-PAGE and detection by immunoblotting with HRP-streptavidin and luminescence readout. Thermo Scientific™ PageRuler™ Plus Prestained Protein Ladder was acquired with white light illumination and automatically merged to the chemiluminescent image (imager Celvin S). (**C**) Side-by-side comparison of concentration data for hTGase 2 (in fmol/µg total protein) in cell lysates from different cell lines determined with the two-site sandwich ELISA, the FA-based assay and the activity-based ELISA. n.d. denotes not determined.

To qualitatively prove that the biotinylated inhibitor **4** is able to selectively label hTGase 2 in cell lysates, recombinant hTGase 2 and selected cell lysates (A375, NCI-H292, and SW480) were treated with **4** and the formed hTGase 2-**4**-complex was detected after separation with SDS-PAGE, followed by immunoblotting (HRP-conjugated streptavidin) and visualization by luminescence readout. Figure 6B clearly shows the occurrence of the expected bands around 80 kDa in mixtures of recombinant enzyme and of cell lysates treated with **4** which are not visible without pretreatment with **4**. It is worth noting that a band with an apparent molar mass slightly lower than hTGase 2 appeared in treated and untreated cell lysates. This band might originate from a naturally biotinylated protein which was cross-detected by the applied HRP-conjugated streptavidin. In this context, biotin-dependent carboxylases harbor biotin covalently bound to a lysine residue [57] with propionyl-CoA carboxylase (PCC) being detected in the investigated cell lines based on its α subunit (PCCA, MW of 80 kDa, Figure S2 in Supplementary Materials).

Encouraged by these results we proceeded with the development of an activity-based ELISA to quantify hTGase 2 in cell lysates (Figure 6A). To this end, lysates of selected cell lines were treated under similar buffer conditions as for the FA measurements with biotinylated inhibitor **4** (20 µM, 30 min at 30 °C). The removal of excess inhibitor was performed by simple precipitation of proteins in acetone [58], which turned out to be more convenient than the filtration of the reaction mixture using Vivaspin^®^ centrifugal concentrators with a molecular weight cut-off of 10 kDa. For quantification, a standard curve with the “hTGase 2-**4**” complex (Figure S3 in Supplementary Materials) was recorded. As shown in Figure 6C, cell lysates of A375, MeWo, NCI-H292, MDA-MB-231 and HUVEC (0.01 g/L) were investigated but the quantification of the amount of TGase 2 was only possible for the latter three as the luminescence signal obtained for A375 and MeWo was not distinguishable from control measurements with **3** instead of **4**. It is worth noting that the activity-based ELISA gave lower but still comparable concentrations of hTGase 2 for NCI-H292, MDA-MB-231 and HUVEC lysates compared to the FA-based assay (e.g., 12.3 versus 21.1 fmol/µg for MDA-MB 231).

### 2.4. Quantification of the Cellular Expression of TGase 2 by a Two-Site Sandwich ELISA

To compare the activity-based TGase 2 concentrations with concentration values for the total TGase 2 protein, we determined the latter concentrations using a commercially available two-site sandwich ELISA (Zedi*Xclusive* Tissue Transglutaminase EIA, E018, Zedira^®^) for selected cell lines (A375, MeWo, NCI-H292, MDA-MB-231, SW480, HUVEC). The conditions for cell lysate preparation, in particular the use of the modified RIPA buffer, as chosen for the activity-based methods were also adopted for the two-site sandwich ELISA. The cell lysate concentration was adjusted to 0.1 g/L and a standard curve was recorded with recombinant hTGase 2 (Figure S4 in Supplementary Materials). The calculated TGase 2 concentrations are shown in Figure 6C in comparison to the data from the activity-based methods. It becomes apparent that the concentration values for the total TGase 2 protein exceed those for activated TGase 2 by factors of 2–4 (ELISA versus FA-based assay), but the relative differences among the investigated cell lines are comparable for data obtained by the different methods.

### 2.5. Analysis of the Cellular Expression of Different Transglutaminases by Western Blot 

**Figure 7:**
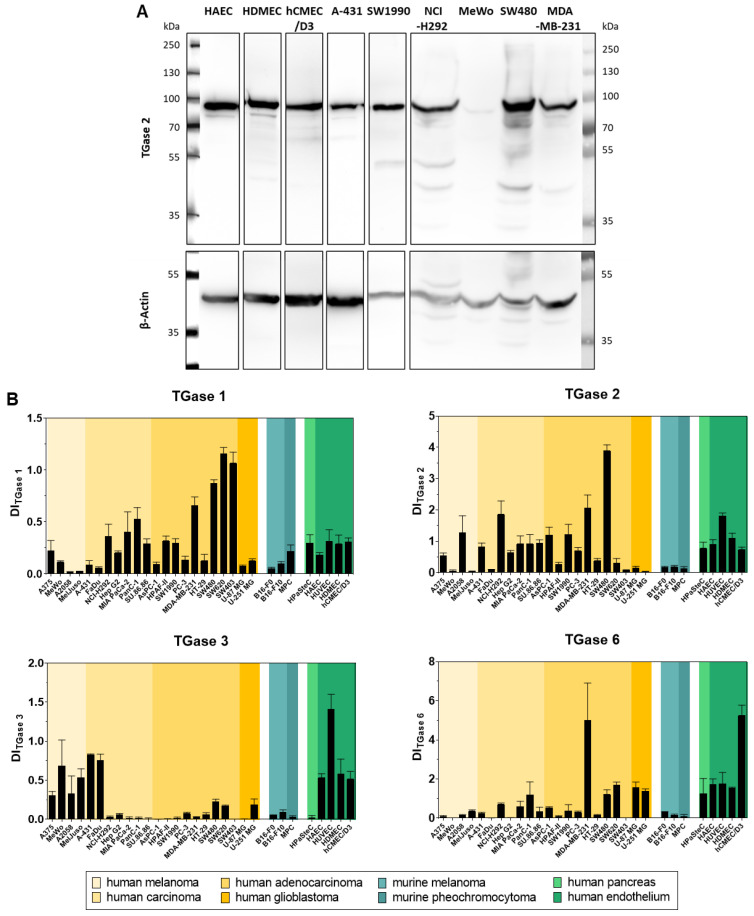
Synthesis of TGase 1, TGase 2, TGase 3, and TGase 6 in different human and mouse cancer cell lines and human noncancer cell lines. (**A**) Exemplary Western blot lanes for selected cell lines which show additional bands of apparent lower molar mass than the full length TGase 2. Thermo Scientific™ PageRuler™ Plus Prestained Protein Ladder was acquired with white light illumination and automatically merged to the chemiluminescent image (imager Celvin S). (**B**) Relative expression of TGases in different cell lines was investigated by Western blotting. Analysis of the blots was performed via densitometry and data were related to β-actin as loading control. n ≥ 3, mean ± SEM. Cell lines were sorted by tumor type and indicated by different colors: human melanoma, carcinoma, adenocarcinoma, glioblastoma (highlighted in yellow), and mouse melanoma and pheochromocytoma origin (highlighted in cyan), as well as human pancreatic and endothelial cell lines (highlighted in green). Densitometric indices, DI, were calculated using Equation (4) (Section 4.6).

In addition to the screening of the panel of cell lines for their TGase 2 activity with the FA-based assay, we assessed the relative expression of TGase 2 and other isoforms by Western blot analysis. The results are shown in Figure 7. While a distinct protein synthesis of TGases 1–3 and 6 was observed in various cell lines, FXIII was only detected in SW480 cells (data not shown). The highest expression values for TGase 1 were observed in the adenocarcinoma cell lines SW480, SW620 and SW403, for TGase 3 in human endothelial cell lines (HAEC, HUVEC, HDMEC, hCMEC/D3), in melanoma cell lines (A375, MeWo, A2058, MelJuso) and in epidermoid (A-431) and squamous cell (FaDu) carcinoma cell lines, for TGase 6 in the breast cancer cell line MDA-MB-231 and in the endothelial cell line hCMEC/D3.

**Figure 8:**
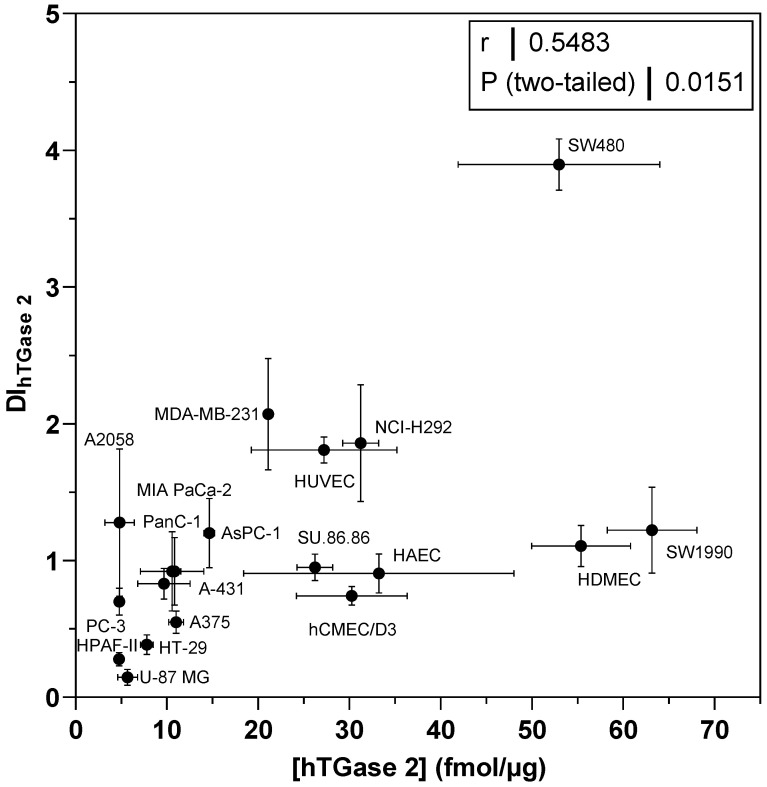
Correlation of relative expression values for hTGase 2 obtained by Western blot analysis with concentrations of active hTGase 2 obtained by the FA-based assay. Data for correlation are taken from Figure 4 (FA-based assay) and Figure 7 (Western blot). Only those cell lines showing hTGase 2 activity above the detection limit (Figure 4A) were considered for correlation.

For TGase 2, the relative expression data in Figure 7B were correlated with the concentration values derived from the FA-based assay (Figure 8). Basically, the higher the relative expression of TGase 2, the higher the measured TGase 2 activity, even though the correlation is only moderate (r = 0.55). For most of the cell lines, a single band corresponding to the molecular weight of TGase 2 (78 kDa) was detected after immunoblotting. However, for a subset of cell lines additional bands corresponding to lower molar masses were observed (Figure 7A), which might indicate the presence of alternative splicing variants or proteolytic products [25,59]. These bands were of negligible intensity compared to the band of the full-length protein and were therefore not considered for the densitometric quantification.

## 3. Discussion

The transamidase activity of TGase 2 in stimulated cells as well as in cell and tissue homogenates is a valuable parameter to compare different cell lines for their TGase 2 expression, to verify the success of the TGM2 gene knock-out, to investigate the impact of sequence mutations on the transamidase activity and, furthermore, to assess the effects of substances which influence enzyme activity. Reliable activity assays are known for this purpose; however, a homogenous and continuous assay format is largely missing. Therefore, we envisaged that our recently developed fluorescence anisotropy (FA)-based assay using DMC and **R-I-Cad** would be a useful tool for studying TGase 2 in biological samples. Generally, in FA-based readouts the degree of polarization of linearly polarized light after passage through a solution containing a suitable fluorophore is measured. The degree of polarization is thereby inversely related to the rotational rate of the fluorophore, which links the measured FA value to the size of the molecule. Therefore, binding of the rather small fluorophore or fluorophore-labeled probe (**R-I-Cad**) to a protein (DMC) results in an increase in the FA value and vice versa [60,61]. In this context, Franklin and Pruneda [62] recently developed a kinetic FA-based assay to follow all stages of protein ubiquitination using recombinant proteins, which highlights that even a cascade of sequential binding and release events can be continuously measured with FA as readout signal. This might not be possible with any other detection method so far.

For the selective detection of TGase 2 in biological samples by substrate-based assays the potential cross-detection of other members of the TGase family, in particular when using DMC as acyl donor, must be considered. The lack of specificity for substrate pairs such as DMC/dansyl-cadaverine [63,64] and DMC/KXD [52,65] has previously been shown, but no quantitative data are available. Therefore, we characterized DMC/**R-I-Cad** for their substrate potential toward selected other recombinant TGases, i.e., hTGases 1, 3, 6, and hFXIIIa. Indeed, these TGases catalyzed the incorporation of **R-I-Cad** into DMC and the substrate potential by means of V_max_/*K*_m_ is even higher for hTGase 1 than for hTGase 2 (Figure 1 and Table 1). Consequently, to record the transamidase activity of TGase 2 in biological samples by means of the FA-based assay, control experiments in the presence of selective inhibitors are mandatory to correctly determine the contribution of TGase 2 to the overall signal.

A puzzling result regarding the characterization of DMC/**R-I-Cad** is the apparent substrate inhibition behavior at high DMC concentration, which is observed for all studied hTGases and also gpTGase 2. This is also the case when using the related rhodamine B-labeled **R-S-Cad** but not for the fluorescein-labeled **F-Cad** [53,54]. An analysis of reaction mixtures containing **R-I-Cad**, DMC and hTGase 2 by SDS-PAGE and in-gel fluorescence detection revealed that the rate of product formation, i.e., formation of DMC covalently labeled with **R-I-Cad**, is indeed reduced at high DMC concentrations (Figure 2A,B). In a previous report, Case and Stein characterized the action of gpTGase 2 and hTGase 2 toward DMC/dansyl-cadaverine in detail and found that substrate inhibition occurs only at high concentrations of dansyl-cadaverine but not at high concentrations of DMC [66]. Therefore, another phenomenon might occur in the case of rhodamine B-labeled cadaverines which causes the reduced product formation. Considering the increasing initial FA values with increasing DMC concentrations (Figure 2C) and the results from the filtration experiments in the presence and absence of DMC (Figure 2D), we concluded that **R-I-Cad** binds noncovalently to DMC which becomes more pronounced at higher concentrations of the protein. This in turn leads to a reduction in the concentration of free **R-I-Cad** and thus, reduces the concentration of the available substrate, which explains the diminished product formation rate at high concentrations of DMC. The susceptibility of **R-I-Cad** to nonspecific protein binding can be attributed to the rhodamine B moiety and was also observed recently for a TAMRA-labeled albumin binder that binds even to denatured albumin while a FAM-labeled analog does not [67]. Obviously, the cationic character of the former ones differentiates the rhodamine dyes from the fluorescein dyes and might be causative for this phenomenon. The finding of **R-I-Cad**’s nonspecific binding has also implications for the application of the FA-based assay in cell lysates. A protein concentration ≤ 1 g/L accounts for negligible nonspecific binding of the fluorescent substrate and ensures the correct quantification of the TGase 2 activity as shown exemplarily for A375 cell lysates with spike/recovery and linearity of dilution experiments (Figure 3E,F, respectively).

The screening of the 30 cancer and noncancer cell lines revealed two key aspects regarding the substrate-based detection of TGase 2 in cell lysates (Figure 4A): (I) the addition of DMC as acyl donor is necessary to achieve an increase in the FA signal indicating that the potential incorporation of **R-I-Cad** into endogenous proteins is not sufficient for signal readout; (II) there is no measurable contribution of other TGases to the overall signal, even though TGases 1, 3, and 6 are expressed in the cell lines (Figure 7B), as the FA increase after preincubation of the cell lysates was abolished in the presence of the selective TGase 2 inhibitor **1**. For 19 out of the 30 cell lines, an FA increase above the background signal was detected, allowing for calculation of the respective hTGase 2 concentration in the range of 4.5 to 63 fmol/µg total protein (Figure 4B). To further validate this result and thus the accuracy of the FA-based assay, we envisaged a complementary activity-based method. Inspired by the classical activity-based protein profiling (ABPP) approach and previously described alkyne-functionalized dihydroisoxazoles [68], we synthesized the biotin-labeled *N*^ε^-acryloyllysine piperazide **4** via CuAAC using the alkyne-functionalized compound **3** (Figure 5A). Even though compound **4** exhibits a rather large modification at the pyridylpiperazine moiety compared to **3** as well as to previously reported *N*^ε^-acryloyllysine piperazides [54,55,69,70], a high inhibitory potency was retained (*k*_inact_/*K*_I_ = 7260 M^−1^s^−1^). Even more importantly, compound **4** (and also **3**) still exhibits an excellent selectivity for hTGase 2 over other members of the TGase family (Figure 5B,C, Table 2). The suitability of compound **4** for targeting hTGase 2 in cell lysates was qualitatively proven by detecting the complex of hTGase 2 and **4** in cell lysates via SDS-PAGE showing the expected band at the molar mass of hTGase 2 after luminescence readout (Figure 6B). For the quantitative determination of the hTGase 2 concentration, an activity-based ELISA was established, which is based on capturing the enzyme-inhibitor complex on streptavidin-coated wells (Figure 6A). The hTGase 2 concentrations derived for NCI-H292, MDA-MB-231 and HUVEC cells were in accordance with the respective values obtained with the FA-based assay, which proves the accuracy of both methods (Figure 6C).

We previously developed an ^18^F-labeled irreversible inhibitor of TGase 2 and quantified the TGase 2 concentration in A375 cells (after stimulation with ionomycin) and A375 cell lysates with radio-SDS-PAGE [71]. For the A375 cell lysate, we determined a hTGase 2 concentration of 0.28 fmol/µg total protein while herein a value of 11.0 fmol/µg total protein was determined using the FA-based assay. This rather large discrepancy presumably originates from the low reaction rate of the radiolabeled inhibitor at concentrations in the double to triple-digit nM range leading to incomplete binding to TGase 2 after the applied reaction time of 25 min and thus, to an underestimation of the TGase 2 concentration.

In addition to the introduction of biotin, the alkyne-functionalized inhibitor **3** can be envisaged for the introduction of other azide-functionalized reporter groups such as fluorophores [72], radiolabeled prosthetic groups [73] or albumin binders [67] by CuAAC. Furthermore, an in situ click chemistry approach, i.e., reaction of compound **3** with TGase 2 followed by coupling of an azide-functionalized reporter group via CuAAC to the protein-bound alkyne appears to be possible [68,74,75].

When assessing the transamidase activity of TGase 2 in cell lysates under conditions optimal for this enzymatic function (high Ca^2+^ concentration and reducing milieu), it is reasonable to assume that there is a correlation between the total and the activity-derived protein concentrations. Such a correlation can be obtained in plasma samples between factor XIIIa activity quantified with a fluorogenic isopeptidase assay and total factor XIII concentration quantified with an antigen assay [76]. To address this assumption herein, we also screened the panel of 30 cell lines for their relative expression of TGase 2 by Western blot analysis (Figure 7B) and correlated the results with the concentration data derived from the FA-based assay for those cell lines exhibiting a measurable TGase 2 activity (Figure 8). Even though the correlation analysis furnished only a moderate correlation coefficient (r = 0.55), there is a general positive correlation between the total and activity-derived protein amount. A crucial aspect that should be mentioned in this context is that the relative expression was assessed by comparing results from different blots. This was unavoidable for the number of investigated cell lines but is generally not recommended [77]. The relative tendency of the activity-derived TGase 2 amount among selected cell lines was also confirmed by protein quantification using a commercially available sandwich ELISA (Figure 6A,C). This in summary allows the conclusion that the activity-based TGase 2 amount in cell lysates is a measure for the total protein amount.

Among the investigated cell lines, six cell lines (MIA PaCa-2, PanC-1, Su.86.86, AsPC-1, HPAF-II, and SW1990) are of pancreatic carcinoma origin. A distinct TGase 2 activity/concentration was measured in lysates of these cells with the FA-based assay and the order from highest to lowest is SW1990 > SU.86.86 > AsPC-1 > MIA PaCa-1 ≈ PanC-1 > HPAF-II (Figure 4). It is worth noting that with the exception of HPAF-II, the TGase 2 activity is significantly higher than the activity measured for lysates of pancreatic stellate cells (HPaSteC). In this context, Verma et al. [78] screened 12 pancreatic ductal adenocarcinoma cell lines including AsPC-1, MiaPaCa-2, HPAF-II, Panc-1, and Su.86.86 for their TGase 2 expression by Western blotting and obtained similar relative tendencies among the cell lines. Furthermore, Su et al. [79] found a higher expression of TGase 2 in SW1990 than in PanC-1 and AsPC-1. Moreover, the increased TGase 2 concentration in pancreatic carcinoma cells compared to normal pancreatic cells determined herein is in line with results from immunostainings of TGase 2 in normal pancreas and pancreatic ductal adenocarcinoma specimens [34]. In contrast to pancreatic stellate cells, lysates of the four human endothelial cell lines (HAEC, HUVEC, HDMEC, and hCMEC/D3) exhibit a high TGase 2 activity/concentration. This is in accordance with previous studies showing the abundant expression of TGase 2 in the endothelium or endothelial cells [80,81,82,83].

In addition to assay methods based on a DMC/amine substrate pair, it should be mentioned that Perez Alea et al. previously developed a colorimetric (but discontinuous) assay for TGase 2 which is based on the TGase 2-catalyzed binding of a biotinylated acyl donor peptide (pepT26) to immobilized spermine (commercially available from Covalab). This assay is largely selective for TGase 2 due to the preferred recognition of pepT26 by TGase 2 [84]. Furthermore, this assay appears to be highly sensitive as a concentration of recombinant hTGase 2 as low as 40 ng/mL can be detected and allows for quantification of TGase 2 in cell lysates. There were also two other FA-based assay methods described for TGase 2 which follow the incorporation of fluorescein-labeled pepT26 (“FL-pepT26”) into BSA (transamidase activity) [85] or the release of this peptide from its conjugate with protein S100A4 (isopeptidase activity) [86]. Both methods might be selective for TGase 2; however, the application for measuring TGase 2 activity in cell lysates has not been demonstrated so far.

Overall, we characterized in detail the FA-based assay with DMC and **R-I-Cad** as substrate pair for the activity-based quantification of TGase 2 in cell lysates and clearly showed the suitability of this homogenous and continuous assay for this purpose. Concomitantly, its limitations were elaborated. A summary of the key aspects for its use with cell lysates complemented by further applications is given in Table 3.

## 4. Materials and Methods

### 4.1. General

The kinetic characterization of compounds **3** and **4** by means of their *k*_inact_/*K*_I_ values was performed using a fluorimetric activity assay (substrate Z-Glu(HMC)-Gly-OH) as described recently [54,56].

Several buffers were used herein, which are listed in the following Table 4. 

### 4.2. Cell culture and Sample Preparation

To analyze the expression of different TGases in lysates and supernatants of cell lines (Table 5) by Western blotting, 1 × 10^6^ cells/well were seeded in 5 mL of the appropriate cell culture medium (Table 5) in a 6-well plate. Cells were cultured for 24 h under standard cell culture conditions (37 °C, saturated water vapor atmosphere with 5% CO_2_ in an incubator). 1 mL of the cell culture supernatant was transferred into an Eppendorf tube and centrifuged for 15 min at 16,000× *g* and 4 °C before 900 µL of the supernatant were removed gently. The remaining 100 µL were mixed with 25 µL 5× SDS-PAGE protein loading buffer, heated to 99 °C for 10 min and frozen for subsequent Western blot analysis. Cells were washed with PBS and detached using 2 mM of EDTA in PBS solution for 30 min. After centrifugation at 300× *g* for 5 min, cell pellets were frozen for later cell lysis.

To analyze the activity of TGase 2 in cell lysates by the FA assay, 2 × 10^6^ cells were seeded in 10 mL of the appropriate cell culture medium in a T75 flask. Cells were cultured until confluence under standard cell culture conditions. Cell culture supernatant was removed, cells were washed with PBS and detached as described above.

### 4.3. Fluorescence Anisotropy Assay

All measurements were conducted at 30 °C over 900 s (interval of 30 or 37 or 48 s) using Synergy 2, Synergy 4 and Cytation 5 multimode microplate readers (BioTek Instruments, Software Gen 5, Winooski, VT, USA) and black 96-well BRANDplates with F-bottom wells (BRAND, Wertheim, Germany). Experiments were conducted at an excitation wavelength of 540 nm and an emission wavelength of 620 nm. The FA (r) was calculated by the Gen 5 software from the measured parallel and perpendicular fluorescence intensities (I_ǁ_ and I_⊥_, respectively) according to Equation (1) using a G factor of 0.87 (preset value) for all microplate readers [53,87].

(1) $$r\;=\frac{I_{||}-\;G\;\times {\;I}_{⊥}}{I_{||}+2G\;\times {\;I}_{⊥}}$$

It should be mentioned that the G factor was not determined by us and certainly varies between the different devices which explains the different absolute FA values. However, according to Banks and Harvey [88], the assay window, which is herein the observed FA rate, “is insignificantly changed by G factor variation”. To prove this for our devices, we measured the FA rate in A375 cell lysates using the Synergy 4 and Cytation 5 microplate readers and found the rates not significantly different (data not shown). All further data analyses including calculation of rates by linear regression of the FA over time, curve fitting, and statistics were conducted with GraphPad Prism (versions 6.0.7 or 9.1.2, GraphPad Software, San Diego, CA, USA). The assay mixture (100 or 200 µL) contained aqueous solution (95 or 190 µL) and DMSO (5%, *v*/*v*, 5 or 10 µL). The concentrations of the enzyme stocks were 0.5 or 1 mg/mL and aliquots were stored at −80 °C. The concentration of **R-I-Cad** was set to 0.74 or 0.81 µM and the respective value is specified for each experiment. According to the previously recorded dependence of the FA rate from the concentration of **R-I-Cad** at 30 µM DMC, the FA rate is constant over a broad concentration range of **R-I-Cad**, i.e., from 0.0024 to 0.81 µM [53].

To provide values of mean and SEM, the corresponding regression analyses were separately accomplished for each experiment, and the obtained fit values were collected and statistically analyzed. The kinetic characterization of the substrate DMC by means of **R-I-Cad** [53] and that of inhibitors **1** and **2** on hTGase 2 (5 min preincubation period) [54] were already performed in previous studies of our group. All TGase isoforms [hTGase 2 (T022), hTGase 1 (T009), hTGase 3 (T013), hTGase 6 (T021), and hFXIIIa (T070)] were purchased from Zedira (Darmstadt, Germany).

#### 4.3.1. Kinetic Characterization of the Acyl Donor DMC toward Human Transglutaminases Using the Acyl Acceptor R-I-Cad

For the kinetic characterization of DMC (Figure 1B) in the presence of a fixed concentration of **R-I-Cad** (0.74 µM), eight different concentrations of DMC (0.3–300 µM) were used (2–3 separate experiments, each performed in duplicate). The corresponding stock solutions of DMC and **R-I-Cad** were prepared in assay buffer A and DMSO, respectively. DMC (50 µL), **R-I-Cad** (5 µL) and DMSO (5 µL) were added to assay buffer A (130 µL) and the mixture was preincubated for 30 min at 30 °C. The reactions were initiated by addition of the respective enzyme stock solution (in enzyme buffer B, 10 µL, 40 µg/mL for hTGase 1 and 100 µg/mL for hTGase 3, hTGase 6, and hFXIIIa) or enzyme buffer B. The recorded time courses of type FA = f(t) were analyzed by linear regression to the experimental data over 900 s. Values for *K*_m_, *K*_i_ and V_max_ were calculated according to the equation of substrate inhibition (Equation (2)) [89].

(2) $$v=\frac{V_{\max}*\left[S\right]}{K_{m}+\left[S\right]*\left(1+\frac{\left[S\right]}{K_{i}}\right)}$$

To investigate the dependence of the enzyme activity on the respective TGase concentration (Figure 1C; 30 μM of DMC and 0.74 μM of **R-I-Cad**), seven and nine different concentrations of hTGase 1/6 (0–5 µg/mL) and hTGase 3/hFXIIIa (0–10 µg/mL) were used, respectively. **R-I-Cad** (5 µL), DMSO (5 µL) and the respective TGase (10 µL) or enzyme buffer B (10 µL) were added to assay buffer A (130 µL) and the mixture was preincubated for 30 min at 30 °C. The reactions were initiated by addition of DMC. The recorded time courses of type FA = f(t) were analyzed by linear regression to the experimental data over 900 s.

To characterize the dependence of the enzyme activity on the hTGase 2 concentration under the conditions for the cell lysate measurements (Figure 3D; 30 μM of DMC and 0.81 μM of **R-I-Cad**), six different concentrations of hTGase 2 (0–5 µg/mL, the active concentration was assumed to be equal to the total protein concentration) were used. Modified RIPA buffer A (50 µL), DMSO (2.5 µL) and hTGase 2 (in enzyme buffer A, 5 µL) or enzyme buffer A (5 µL) were added to assay buffer B (15 µL) and the mixture was preincubated for 10 min at 30 °C. The reactions were initiated by addition of **R-I-Cad** (2.5 µL) and DMC (25 µL, stock in assay buffer B). The recorded time courses of type FA = f(t) were analyzed by linear regression to the experimental data over 420 s.

#### 4.3.2. Characterization of Irreversible Inhibitors toward Transglutaminases

For the characterization of the irreversible inhibitors **1**–**4** toward the different TGases at fixed preincubation times of 5 or 30 min (Figure 5B,C), 10–12 different concentrations of the inhibitors were used (three separate experiments, each performed in duplicate). The appropriate stock solutions of the inhibitors were prepared in DMSO. For all TGases, the concentrations of DMC and **R-I-Cad** were set to 30 and 0.74–0.81 µM, respectively. Inhibitor (5 µL), **R-I-Cad** (5 µL) and the respective TGase (in enzyme buffer B, 10 µL, 40 µg/mL for hTGase 1, 40 or 100 µg/mL for hTGase 2, and 100 µg/mL hTGase 3, hTGase 6, and hFXIIIa) were added to assay buffer A (130 µL) and the mixture was incubated for 5 or 30 min at 30 °C. The reactions were initiated by addition of DMC (50 µL, stock in assay buffer A). The recorded time courses of type FA = f(t) were analyzed by linear regression to the experimental data over 900 s. The inhibitor concentration, [I], causing 50% inhibition, IC_50_, and the Hill slope, n_H_, were calculated according to Equation (3),

(3) $$\mathrm{rate}\;=\;\mathrm{Bottom}\;+\frac{\left(\mathrm{Top}\;-\;\mathrm{Bottom}\right)\times {\left[I\right]}^{\mathrm{nH}}}{{\left[I\right]}^{\mathrm{nH}}+{\;\mathrm{IC}}_{50}{}^{\mathrm{nH}}}\;$$

with Bottom and Top representing the lower and upper plateaus of the sigmoidal dose–response curve, respectively.

#### 4.3.3. Characterization of TGase 2 Activity in Cell Lysates

Lysis of cell pellets was performed as described recently [54,71]. Accordingly, cell pellets were resuspended in 50 µL modified RIPA buffer A, incubated on ice for 30 min and applied to ultrasound (Bandelin Sonopuls, 2 × 15 s, 20% power at ambient temperature). Subsequently, cell lysates were incubated on ice for 10 min and were then spun for 15 min at 4 °C at 15,000× *g*. The supernatants were transferred into fresh tubes. The protein content was determined with a BCA protein assay (ThermoFisher, Waltham, MA, USA) according to the manufacturer’s protocol. Lysates were stored at −60 °C.

For the determination of TGase 2 activity in cell lysates (Figure 4A), the volume of the assay mixture was reduced to 100 µL compared to 200 µL for measurements using the recombinant enzymes. For control measurements, inhibitor **1** (10 µM) was used or DMC was omitted. Assay buffer B (20 µL) and DMSO (2.5 µL) or inhibitor **1** (2.5 µL of 0.4 mM stock in DMSO) were added to the solution of cell lysate (50 µL, a maximum final concentration of 1 g/L is recommended, see the Discussion section) and the mixture was incubated for 10 min at 30 °C. The reactions were initiated upon addition of **R-I-Cad** (2.5 µL) and DMC (25 µL, stock in assay buffer B) or assay buffer B (25 µL), respectively. The recorded time courses of type FA = f(t) were analyzed by linear regression to the experimental data over 300 or 900 s depending on the shape of the curve.

Conversion of the obtained FA rate (mA/s) into concentration (fmol/µg total protein) was achieved using the black standard curve shown in Figure 3A and the molar mass of hTGase 2 (78,000 g/mol for T022, Zedira^®^). For measurements in which a lysate concentration of 1.5 or 2 g/L was used, the FA rates were multiplied with a factor of 1.8 (see Discussion for details).

#### 4.3.4. Separation and Visualization of R-I-Cad-Labeled DMC by SDS-PAGE and in-Gel Fluorescence Measurement

The assay setting followed the description given in Section 4.3.1, but with altered DMC concentrations being used (0 and 1–300 µM). The final hTGase 2 concentration was 2.5 µg/mL. After incubation for 15 min (i.e., the usual measurement period), 100 µL of each reaction mixture was added to a mixture of 20 µL EDTA (100 mM) and 20 µL 5× SDS-PAGE protein loading buffer. The samples were heated at 99 °C for 5 min, and an aliquot (25 μL) of the mixture was applied to the 10% acrylamide gel. In-gel fluorescence readout was performed using the Amersham Typhoon 5 biomolecular imager (Cy3 filter; excitation at 523 nm; emission range: 560–580 nm).

#### 4.3.5. Assessing Noncovalent Binding of R-I-Cad to DMC with Ultrafiltration

**R-I-Cad** (25 µL, final 8.1 µM) was added to assay buffer A (350 µL) followed by the addition of DMC (125 µL, 300 µM final). A separate mixture was prepared without the addition of DMC (475 µL assay buffer A). To assess the filter binding of **R-I-Cad**, an aliquot (50 µL) of the second mixture was withdrawn before filtration. Both mixtures were transferred to Vivaspin^®^ 500 centrifugal concentrators (3000 MWCO, PES, Sartorius), pre-washed with assay buffer A (2 × 500 µL, 30 °C, 10 min at 10,000× *g*, Hettich UNIVERSAL 320 R centrifuge), and they were centrifuged for 90 min (30 °C, 15,000× *g*). Aliquots (50 µL) of the eluates were withdrawn and analyzed by RP-HPLC (Section 4.8.2).

### 4.4. Visualization of the hTGase ***2***–***4*** complex after SDS-PAGE and immunoblotting

For a standard curve, the hTGase **2**–**4** complex was generated by mixing hTGase 2 (10 µL of 1 mg/mL, final 0.2 mg/mL, the active concentration was assumed to be equal to the total protein concentration) with HEPES buffer A (38 µL) and inhibitor **4** (2 µL of 0.5 mM stock in 25% DMSO/water, final 20 µM and 1% DMSO) in a 1.5 mL Eppendorf tube for 30 min at 30 °C. In order to reach a final concentration of 50 ng/µL, 25 µL of the incubation solution were diluted with 75 µL modified RIPA buffer B. Then, a serial dilution (2.5, 5, 25 ng/µL) was prepared with modified RIPA buffer B. Cell lysates were diluted to 6 g/L with modified RIPA buffer B and 25 µL of diluted lysate were incubated for 30 min at 30 °C with HEPES buffer A (23 µL) and with inhibitor **4** (2 µL of 0.5 mM stock in 25% DMSO/water, final 20 µM and 1% DMSO) and for control with HEPES buffer A alone (25 µL). Then, 5 µL 5× SDS-PAGE protein loading buffer was added to 20 µL standard and cell lysate samples before they were heated up to 99 °C for 10 min. SDS-PAGE using 10% acrylamide gels was run with 25 µL sample per lane for 20 min at 100 V and further 60 min at 140 V. Afterwards, protein transfer to a PVDF membrane was performed by tank blotting for 16 h at 30 V and 45 mA. Membranes were incubated with Western Blot Signal Enhancer (Thermo Fisher Scientific, 21050) according to the manufacturer’s instructions, before they were blocked for 1 h at room temperature in a solution of 1% bovine serum albumin (*w/v*) in TBS-T buffer (10 mM Tris pH 8.0, 150 mM NaCl, 0.05% Tween 20 (*v/v*)), followed by an incubation for 1 h at room temperature with streptavidin-HRP (Thermo Fisher Scientific, 21130), diluted 1:50,000 in blocking solution. Membranes were washed 3 × 20 min with TBS-T buffer. A 1:2 mixture of SuperSignal™ West Pico PLUS Chemiluminescent Substrate (Thermo Fisher Scientific, 34580) and SuperSignal™ West Femto Maximum Sensitivity Substrate (Thermo Fisher Scientific, 34095) was used for band detection with the chemiluminescence imager Celvin S (Biostep).

### 4.5. Activity-Based ELISA Using Biotinylated Inhibitor 4

For a standard curve, the hTGase 2–**4** complex was generated by mixing hTGase 2 (10 µL of 1 mg/mL, final 0.2 mg/mL, the active concentration was assumed to be equal to the total protein concentration) with HEPES buffer A (38 µL) and inhibitor **4** (2 µL of 0.5 mM stock in 25% DMSO/water, final 20 µM and 1% DMSO) in a 1.5 mL Eppendorf tube for 30 min at 30 °C. Afterwards, the hTGase 2–**4** complex was precipitated by adding ice-cold acetone (1 mL) and incubating the mixture for 5 min on ice [58]. The mixture was centrifuged for 10 min at 10,000× *g* at 4 °C. The supernatant containing unbound **4** was removed and the precipitate was resuspended in HEPES buffer B (200 µL) to reach a final concentration of 50 ng/µL. Then, a serial dilution (0, 0.005, 0.01, 0.05, 0.1, 0.5, 1, 5, 10 ng/µL) was prepared with ELISA washing buffer (PBS, pH 7.4, 0.05% Tween 20 (*v/v*) and 0.1% BSA (*w/v*)).

To quantify activatable hTGase 2 in lysates from A375, MeWo, MDA-MB231, NCI-H292 and HUVEC cells, all lysates were diluted to 1 g/L with modified RIPA buffer A. Afterwards, modified RIPA buffer A (16.5 µL), HEPES buffer A (76 µL) and inhibitor **4** (4 µL of 0.5 mM stock in 25% DMSO/water, final 20 µM and 1% DMSO) were added to the lysates (3.5 µL, 0.035 g/L during incubation). The incubation and precipitation of the TGase 2–**4** complex with acetone was conducted as described above. The precipitate was resuspended using an ultrasonic finger (Bandelin Sonopuls, 2 × 15 s, 20% power at ambient temperature) in ELISA washing buffer (350 µL) to adjust a final protein concentration of 0.01 g/L.

To measure lysate specific background signal, cell lysates were also incubated with inhibitor **3**. The procedure follows the steps described for incubations with inhibitor **4** (see above).

Pierce^®^ Streptavidin High Binding Capacity Coated 96-well plates (Thermo Fisher, 15500) were pretreated by washing the wells with ELISA washing buffer (200 µL, 3 × 5 min). Afterwards, the respective samples (100 µL each) were filled into the wells and the plate was sealed with a foil (Fasson^®^ laminated foils). The hTGase 2–**4** complex was allowed to bind to the immobilized streptavidin for around 16–18 h at 25 °C. The next day, the solution was removed by forcefully inverting the plate followed by putting it on a paper towel to dry. Then, the plate was washed again with ELISA washing buffer (200 µL, 3 × 5 min). Thereafter, primary mouse anti-TG2 antibody (100 µL, 1:1000, abcam ab2386) was added and incubation was conducted for 1 h at room temperature followed by a further washing step (200 µL, 3 × 5 min). Subsequently, secondary anti-mouse-IgG-HRP antibody (200 µL, 1:200,000; Sigma A9044) was added for 45 min, followed again by the washing procedure with ELISA washing buffer (200 µL, 3 × 5 min). Finally, Super Signal ELISA Femto solution (100 µL, equal volumes of solution 1 and solution 2, Thermo Fisher, 37074) was added per well in the dark by a multichannel pipette and immediately put into the plate reader (Cytation 5 Cell Imaging Multimode Reader, BioTek). The plate was shaken for 10 s and the luminescence was measured over 30 min at ambient temperature (interval of 75 s). The standard curve was recorded twice and on different days, giving comparable results.

Data analysis was performed using GraphPad Prism (version 9.1.2). To obtain the standard curve, the maximal luminescence value was determined for each progress curve and plotted against the logarithmically transformed amount of hTGase 2–**4** complex. The maximal luminescence values for the sample measurements were also determined and registered within the same data table in Prism (but without defined x values). For the cell lysates samples, luminescence values obtained with **4** were corrected for the respective background luminescence values obtained with **3**. Then, the implemented analysis method “Interpolate a standard curve” with the model “Sigmoidal, 4PL, X is log(concentration)” was chosen to calculate the hTGase 2 amount (in ng) in the cell lysate samples. Conversion of these values into concentrations was achieved using the molar mass of hTGase 2 (78,000 g/mol for T022, Zedira^®^) and the applied amount of cell lysate (1 µg).

### 4.6. Cellular Expression of Different TGases and Propionyl-CoA Carboxylase

Cell pellets were collected in 100 µL RIPA lysis buffer. Cell lysis, SDS-PAGE and Western blotting were performed as described previously [90]. An amount of 25–50 µg total protein was applied per lane. Blots were incubated with primary and secondary antibodies, listed in Table 6.

Western blot images were evaluated using densitometric analysis performed via 1D gel analysis software TotalLab (version 14.0, TotalLab) with a minimum profile background subtraction and automatic band detection (minimum slope 350, noise reduction 5%, 3% of maximum peak). The resulting values for the area under the curve (AUC) were used to calculate the densitometric index for each protein of interest (DI_protein of interest_) for each sample using Equation (4)

(4) $${\mathrm{DI}}_{\mathrm{protein}\;\mathrm{of}\;\mathrm{interest}}=\frac{{\;\mathrm{AUC}}_{\mathrm{protein}\;\mathrm{of}\;\mathrm{interest}}}{{\mathrm{AUC}}_{\beta -\mathrm{actin}}}$$

### 4.7. Two-Site Sandwich ELISA for Quantifying hTGase 2

hTGase 2 in cell lysates was quantified by application of ZediXclusive Tissue Transglutaminase EIA (Zedira, E018) according to the manufacturer’s instructions. Cell lysates were diluted to a concentration of 0.1 g/L with the provided sample buffer and were measured in duplicates. Absorbance at 450 nm was read with a Cytation 5 imaging reader from Biotek. Resulting absorbance values were plotted as a function of the logarithmically transformed concentration values of hTGase 2 and a nonlinear regression was performed using the model of “sigmoidal, 4PL, X is log(concentration)”, as implemented in GraphPad Prism (version 9.1.2).

### 4.8. Chemistry

#### 4.8.1. General

All commercial reagents and solvents were used without further purification unless otherwise specified. Nuclear magnetic resonance spectra were recorded on an Agilent Technologies 400 MR spectrometer consisting of 400/54 premium compact magnet, 400 MR console and 400 MHz OneNMRProbe PT probe head (400 MHz for ^1^H and 101 MHz for ^13^C) or Agilent Technologies 600 spectrometer consisting of 600/54 premium compact magnet, DD2 console and 600 MHz OneNMRProbe PT probe head (600 MHz for ^1^H and 151 MHz for ^13^C). Spectra were processed by using the program MestreNova (version 14.2.1-27684). NMR chemical shifts were referenced to the residual solvent resonances relative to tetramethylsilane (TMS; ^1^H and ^13^C). Mass spectra (ESI) were obtained on a Waters Xevo TQ-S mass spectrometer driven by the Mass Lynx software.

#### 4.8.2. Chromatography

Thin-layer chromatography (TLC) was performed on Merck silica gel F-254 aluminum plates with visualization under UV (254 nm). Preparative column chromatography was carried out on the Flash Chromatography “Selekt System” from Biotage^®^ using appropriate “Sfär” columns and solvent mixtures. The HPLC system used was a LC-20A Prominence HPLC by Shimadzu, consisting of a degasser unit DGU-20A5R; two separate pumping units, LC-A20R; a sample manager, SIC-20ACHT; a column oven, CTO-20AC; a PDA-detector, SPD-M20A; a communication-bus module, CBM-20A; and a fraction collector, FRC-10A. A pair of Phenomenex Jupiter Proteo C18 columns (250 × 4.6 mm; 250 × 21.2 mm, 4 µm, 90 Å) were used as the stationary phases for analytical and preparative RP-HPLC (for compound **4**), respectively. A binary gradient system of 0.1% CF_3_COOH/water (solvent A) and 0.1% CF_3_COOH/CH_3_CN (solvent B) at a flow rate of 1 mL/min (analytical) or between 10 and 20 mL/min (preparative) served as the eluent. For UPLC-DAD-MS, a system from Waters (ACQUITY UPLC I class system including an ACQUITY UPLC PDA e λ detector coupled to a Xevo TQ-S mass spectrometer) was used. An ACQUITY UPLC BEH C18 column (1.7 µm, 130 Å, 100 × 2.1 mm, equipped with an ACQUITY UPLC BEH C18 VanGuard Pre-column, 1.7 µm, 130 Å, 5 × 2.1 mm) was used as stationary phase. A binary gradient system of 0.1% CH_3_COOH/water (solvent A) and 0.1% CH_3_COOH in CH_3_CN/CH_3_OH (1:1, *v/v*, solvent B) at a flow rate of 0.4 mL/min served as the eluent. Lyophilization was performed using the freeze-dryer Alpha 2–4 LSCplus (Christ).

The synthetic procedures and analytical data for compounds **1** and **2** have been described previously [54]. The synthesis of compounds **3** and **4** follows the general synthesis steps for *N*^ε^-acryloyllysine piperazides as recently described by our group [54] and as shown in (Scheme S1 in Supplementary Materials).

#### 4.8.3. Synthesis of 4-Boc-1-(6-ethinylpyridin-2-yl)piperazine (**5**)


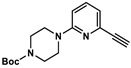


4-Boc-1-(6-bromopyridin-2-yl)piperazine (500 mg, 1.5 mmol, 1 eq.), CuI (14 mg, 0.07 mmol, 0.05 eq.) and Pd(PPh_3_)_4_ (84 mg, 0.07 mmol, 0.05 eq.) was weighed into a round-bottom flask and placed in an inert atmosphere by evacuating and purging the vessel with argon several times. Next, THF (5 mL), trimethylsilylacetylene (TMSA, 312 µL, 2.2 mmol, 1.5 eq.) and diisopropylamine (554 µL, 4 mmol, 2.7 eq.) were added and the reaction mixture was sealed and stirred for 17 h at room temperature. Afterwards, the solvent was removed in vacuo and the residue was taken up in ethyl acetate (20 mL). The organic phase was washed with water (1 × 10 mL), 0.03 M sodium diethyldithiocarbamate/water (5 × 10 mL), and brine (1 × 10 mL), dried over Na_2_SO_4_ and evaporated.

For the removal of the TMS group, the crude product was dissolved in CH_2_Cl_2_ (10 mL) followed by the addition of tetrabutylammonium fluoride (TBAF, 1.20 g, 4.5 mmol, 3 eq.). This mixture was stirred for 35 min. Afterwards, the organic phase was extracted with water (40 mL) and separated. The aqueous phase was washed with CH_2_Cl_2_ (3 × 40 mL). The combined organic phases were dried over Na_2_SO_4_ and evaporated. The crude product was purified via column chromatography (gradient from ethyl acetate-hexane 7:93 to 60:40). The product-containing fractions were combined and evaporated to afford **5** (366 mg, 65%) as a yellowish solid. *R*_f_ 0.4 (ethyl acetate-hexane 1:2); **^1^H NMR** (400 MHz, CDCl_3_) δ = 7.45 (dd, ^3^*J* = 8.6, 7.3 Hz, 1H, H-4 of pyridine), 6.86 (d, ^3^*J* = 7.3 Hz, 1H, H of pyridine), 6.64 (d, ^3^*J* = 8.6 Hz, 1H, H of pyridine), 3.58–3.51 (m, 8H, 4 × CH_2_ of piperazine), 3.06 (s, 1H, CH of alkyne), 1.48 (s, 9H, 3 × CH_3_ of Boc); **^13^C NMR** (101 MHz, CDCl_3_) δ = 154.96 (C=O of Boc), 137.90 (C-4 of pyridine), 117.75 (CH of pyridine), 107.79 (CH of pyridine), 80.16 (quart. C of Boc), 45.10 (4 × CH_2_ of piperazine), 28.58 (3 × CH_3_ of Boc), signals for C-2,6 of pyridine and for the alkyne group are not visible; MS (ESI^+^): *m*/*z* calculated for C_16_H_21_N_3_O_2_: 288.17 [M+H]^+^; found 288.1.

#### 4.8.4. Synthesis of 6-ethinylpyridin-2-yl)piperazine (**6**)


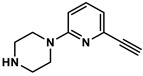


Compound **5** (366 mg, 1.3 mmol) was dissolved in CH_2_Cl_2_ (3 mL). Subsequently, TFA (3 mL) was added slowly under stirring. The reaction mixture was stirred for additional 2 h and the volatile components were removed in a N_2_ stream under reduced pressure (300 mbar). The residue was dissolved in CH_2_Cl_2_ (30 mL) and the organic phase was washed with 0.1 M NaOH (15 mL). The alkaline solution was extracted with CH_2_Cl_2_ (6 × 30 mL). The organic phases were combined, dried over Na_2_SO_4_, and evaporated to afford the respective free amine **6** (216 mg, 91%) as a yellowish solid. **^1^H NMR** (600 MHz, DMSO-*d*_6_) δ = 7.49 (dd, ^3^*J* = 8.7, 7.1 Hz, 1H, H-4 of pyridine), 6.82 (d, ^3^*J* = 8.6 Hz, 1H, H-5 of pyridine), 6.77 (d, ^3^*J* = 7.1 Hz, H-3 of pyridine), 4.09 (s, 1H, CH of alkyne), 3.41–3.34 (m, 4H, 2 × CH_2_ of piperazine), 2.74 (t, ^3^*J* = 5.1 Hz, 4H, 2 × CH_2_ of piperazine). Signal for NH is not visible; **^13^C NMR** (151 MHz, DMSO-*d*_6_) δ = 158.95 (C-2 of pyridine), 139.39 (C-6 of pyridine), 137.75 (C-4 of pyridine), 116.32 (C-5 of pyridine), 107.53 (C-3 of pyridine), 83.93 (C of alkyne), 78.33 (CH of alkyne), 45.57 (2 × CH_2_ of piperazine), 45.38 (2 × CH_2_ of piperazine); MS (ESI^+^): *m*/*z* calculated for C_11_H_13_N_3_ = 188.12 [M+H]^+^; found 188.1.

#### 4.8.5. Synthesis of N^α^-Boc-N^ε^-Acryloyl-l-lysine-4-(6-ethinylpyridin-2-yl)piperazide (**7**)


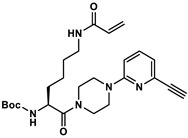


To a solution of *N*^α^-Boc-*N*^ε^-acryloyl-l-lysine (346 mg, 1.15 mmol, 1 eq., synthesized as previously described [54]) and DIPEA (324 μL, 1.86 mmol, 1.6 eq.) in THF (5 mL) was added **6** (216 mg, 1.15 mmol, 1 eq.). Subsequently, HATU (526 mg, 1.38 mmol, 1.2 eq.) was added and the reaction mixture was stirred for 1 h. The solvent was removed in vacuo, and the residue was dissolved in CH_2_Cl_2_ (40 mL). The organic phase was washed with a mixture of saturated NaHCO_3_ and brine (15 mL, 1:1). The aqueous phase was extracted with CH_2_Cl_2_ (2 × 50 mL). The organic phases were combined, dried over Na_2_SO_4_, and evaporated. The crude product was purified via column chromatography (gradient from acetone-CH_2_Cl_2_ 5:95 to 40:60). The product-containing fractions were combined and evaporated to afford **7** (586 mg, >99%) as a yellow, oily residue. *R*_f_ 0.16 (acetone-CH_2_Cl_2_ 20:80); MS (ESI^+^): *m*/*z* calculated for C_25_H_35_N_5_O_4_ = 470.28 [M+H]^+^; found 470.2.

#### 4.8.6. Synthesis of N^ε^-Acryloyl-l-lysine-4-(6-ethinylpyridin-2-yl)piperazide (**8**)


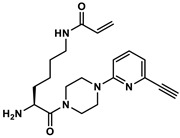


The removal of the Boc group from compound **7** (586 mg) was accomplished as described for compound **5** (Section 4.8.4). Compound **8** (367 mg 80%) was obtained as a yellow, oily solid. MS (ESI^+^): *m*/*z* calculated for C_20_H_27_N_5_O_2_ = 370.22 [M+H]^+^; found 370.0.

#### 4.8.7. Synthesis of N^α^-Phenylacetyl-N^ε^-acryloyl-l-lysine-4-(6-ethinylpyridin-2-yl)piperazide × TFA (**3**)


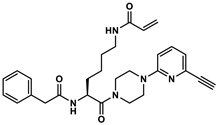


To a solution of **8** (367 mg, 1 mmol, 1 eq.) and TEA (277 μL, 2 mmol, 2 eq.) in CH_2_Cl_2_ (3 mL) was added phenylacetyl chloride (131 μL, 1 mmol, 1 eq.). The reaction mixture was stirred for 1 h. Afterward, CH_2_Cl_2_ (20 mL) was added and the organic phase was washed with a mixture of saturated NaHCO_3_ and brine (20 mL, 1:1). The aqueous phase was extracted with CH_2_Cl_2_ (3 × 20 mL). The organic phases were combined, dried over Na_2_SO_4_, and evaporated. The crude product was purified via column chromatography (gradient from ethanol-CH_2_Cl_2_ 1:99 to 10:90). The product-containing fractions were combined and evaporated to afford **3** (246 mg, 51%) as a yellowish solid. *R*_f_ 0.31 (ethanol-CH_2_Cl_2_ 5:95); **^1^H NMR** (400 MHz, CDCl_3_) δ = 7.48 (dd, ^3^*J* = 8.5, 7.4 Hz, 1H, H-4 of pyridine), 7.38–7.27 (m, 5H, CH of phenyl), 6.90 (d, ^3^*J* = 7.3 Hz, 1H, H-3,5 of pyridine), 6.65 (d, ^3^*J* = 8.6 Hz, 1H, H-3,5 of pyridine), 6.53 (d, ^3^*J* = 8.0 Hz, 1H, N_α_H), 6.25 (dd, ^3^*J* = 17.0, ^2^*J* = 1.5 Hz, 1H, CH*H* of acryloyl), 6.05 (dd, ^3^*J* = 17.0, 10.2 Hz, 1H, CH of acryloyl), 5.92 (broad s, 1H, N_ε_H), 5.59 (dd, ^3^*J* = 10.3, ^2^*J* =1.5 Hz, 1H, C*H*H of acryloyl), 4.94 (td, ^3^*J* = 8.4, 1H, C_α_H), 3.79–3.49 (m, 10H, 4×CH_2_ of piperazine, CH_2_-phenyl), 3.31–3.25 (m, 2H, C_ε_H_2_ of lysine), 3.09 (s, 1H, CH of alkyne), 1.79–1.47 (m, 4H, C_β_H_2_ and C_δ_H_2_ of lysine), 1.36–1.28 (m, 2H, C_γ_H_2_ of lysine); **^13^C NMR** (101 MHz, CDCl_3_) δ = 171.12 (CO), 170.40 (CO), 165.87 (CO), 138.93 (C-4 of pyridine), 134.69 (C-1 of phenyl), 131.09 (CH of acryloyl), 129.45 (2 × CH of phenyl), 129.13 (2 × CH of phenyl), 127.57 (C-4 of phenyl), 126.27 (CH_2_ of acryloyl), 118.44 (C-3/5 of pyridine), 108.62 (C-3/5 of pyridine), 48.49 (C_α_H of lysine), 45.15 (CH_2_ of piperazine), 43.87(CH_2_-phenyl), 41.81(CH_2_ of piperazine), 39.14 (C_ε_ of lysine), 32.90 (C_β_ of lysine), 28.53 (C_δ_ of lysine), 22.27 (C_γ_H_2_ of lysine). Signals for the alkyne and C-2,6 of pyridine are not visible; MS (ESI^+^): *m*/*z* calculated for C_28_H_33_N_5_O_3_ = 488.27 [M+H]^+^; found 488.2 [M+H]^+^.

#### 4.8.8. Synthesis of N-(2-(2-(2-(2-(4-(6-(4-(N^6^-acryloyl-N^2^-(2-phenylacetyl)-l-lysyl)piperazin-1-yl)pyridin-2-yl)-1H-1,2,3-triazol-1-yl)ethoxy)ethoxy)ethoxy)ethyl)-5-((3aS,4S,6aR)-2-oxohexahydro-1H-thieno[3,4-d]imidazol-4-yl)pentanamide × 2TFA (**4**)


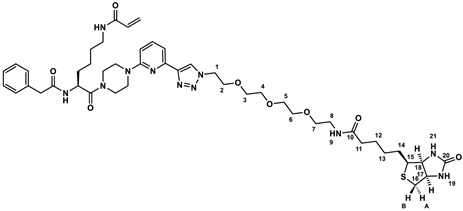


For the copper-catalyzed azide/alkyne cycloaddition, a solution of alkyne **3** (5 mg, 8.3 µmol, 1 eq.) and biotin-PEG3-azide (3.7 mg, 8.3 µmol, 1 eq.) in *tert*-butanol (1 mL) was prepared. Next, an aqueous solution of THPTA (50 μL of 0.1 M solution, 0.6 eq.) was mixed with an aqueous solution of CuSO_4_ × 5 H_2_O (50 μL of 0.1 M solution, 0.6 eq.) and the resulting mixture was added to the *tert*-butanol solution. Afterward, a freshly prepared aqueous solution of sodium ascorbate (50 μL of 1 M solution, 6 eq.) was added and the reaction mixture was stirred for 3 h. Subsequently, a mixture of CH_3_CN/water (3 mL, 1:3) was added and the crude product was purified by RP-HPLC. The product-containing fractions were combined and lyophilized to afford **4** (4.9 mg, 51%) as a white solid.

**^1^H NMR** (400 MHz, DMSO-*d*_6_) δ = 8.48 (s, 1H, H-4 triazole), 8.41 (d, J = 8.2 Hz, 1H, N_α_H of lysine), 8.08–8.00 (m, 1H, N_ε_H of lysine), 7.78 (t, ^3^*J* = 5.6 Hz, 1H, H-9), 7.64 (t, ^3^*J* = 8.0 Hz, 1H, H-4 of pyridine), 7.33 (d, ^3^*J* = 7.4 Hz, 1H, H-5 of pyridine), 7.29–7.22 (m, 4H, H-2,3,5,6 of phenyl), 7.21–7.13 (m, 1H, H-4 of phenyl), 6.76 (d, ^3^*J* = 8.6 Hz, 1H, H-3 of pyridine), 6.41 (broad s, 2H, H-19,21), 6.18 (dd, ^3^*J* = 17.0, 10.0 Hz, 1H, CH of acryloyl), 6.04 (dd, ^3^*J* = 17.1 Hz, ^2^*J* = 2.2 Hz, 1H, C*H*H of acryloyl), 5.53 (dd, ^3^*J* = 10.1, ^2^*J* = 2.4 Hz, 1H, CH*H* of acryloyl), 4.77–4.68 (m, 1H, C_α_H), 4.58 (t, ^3^*J* = 5.3 Hz, 2H, H-1), 4.32–4.26 (m, 1H, H-17), 4.14–4.08 (m, 1H, H-18), 3.91–3.85 (m, 2H, H-2), 3.68–3.30 (m, 20H, 4×CH_2_ of piperazine, CH_2_-phenyl, H-3,4,5,6,7), 3.18–3.03 (m, 5H, H-8,15, C_ε_H_2_ of lysine), 2.80 (dd, ^2^*J* = 12.4, ^3^*J* = 4.9 Hz, 1H, H-16A), 2.57 (d, ^2^*J* = ^3^*J* = 12.0 Hz, 1H, H-16B) 2.04 (t, ^3^*J* = 7.5 Hz, 2H, H-11), 1.74–1.17 (m, 12H, C_β_H_2_, C_γ_H_2_, C_δ_H_2_ of lysine, H-12,13,14). **^13^C NMR** (101 MHz, CDCl_3_) δ = 174.05 (CO), 171.98 (CO), 171.03 (CO), 166.37 (CO), 158.03 (CO), 139.70 (C-4 of pyridine), 134.65 (C-1 of phenyl), 130.86 (CH of acryloyl), 129.52 (2 × CH of phenyl), 129.01 (2 × CH of phenyl), 127.50 (C-4 of phenyl), 126.74 (CH_2_ of acryloyl), 124.06 (C-4 of triazole), 110.91 (C-5 of pyridine), 107.33 (C-3 of pyridine), 77.36, 70.85, 70.58, 70.40, 70.19, 69.78, 69.48, 62.48 (C-18), 60.83 (C-17), 55.52 (C-15), 50.74 (C-1), 49.00 (C_α_ of lysine), 45.66, 45.56, 45.50, 43.45, 42.03, 40.57, 39.52, 39.12, 35.41, 32.23, 31.07, 28.53, 28.02, 27.97, 25.45, 22.47; signals for C-2,6 of pyridine and C-5 of triazole are not visible; assignments of the ^1^H NMR spectrum and the ^13^C NMR spectrum for the biotin moiety were conducted according to literature data [91]; MS (ESI^+^): *m*/*z* calculated for C_46_H_66_N_11_O_8_S = 932.48 [M+H]^+^; found 931.8 [M+H]^+^.

## Figures and Tables

**Table 1:** Kinetic parameters of the substrate DMC for its TGase-catalyzed reaction with **R-I-Cad**.

	hTGase 1	hTGase 2	hTGase 3	hTGase 6	hFXIIIa
V_max_ (mA mL s^−1^ mg^−1^)	121 (13)	26.4 (2.8)	5.80 (0.09)	27.1 (4.1)	16.0 (1.1)
*K*_m_ (µM)	8.77 (1.70)	2.78 (0.10)	6.29 (1.52)	16.6 (4.7)	17.5 (0.7)
*K*_i_ (µM)	270 (72)	174 (16)	571 (53)	189 (61)	135 (14)
V_max_/*K*_m_ (mA mL s^−1^ mg^−1^ µM^−1^)	13.8	9.50	0.92	1.63	0.91

Data shown are mean values ± SEM of 2–3 separate experiments, each performed in duplicate. Data were obtained by nonlinear regression of plots v = f([DMC]) according to substrate inhibition (Equation (2), Section 4.3.1). Data for hTGase 2 have previously been published [54].

**Table 2:** Summary of inhibition parameters of compounds **1–4** toward different human transglutaminases.

	IC_50_ (nM) ^a^						*k*_inact_/*K*_I_ (M^−1^s^−1^) ^b^
	Time (min)	hTGase 1	hTGase 2	hTGase 3	hTGase 6	hFXIIIa	hTGase 2
**1**	5	n.d.	310 ^c^	n.d.	n.d.	n.d.	4880 (20)^c^
	30	>30,000	40 (12)	>30,000	>30,000	>30,000	
**2**	5	n.d.	90 ^c^	n.d.	n.d.	n.d.	8460 (710) ^c^
	30	8990 (1180)	42 (15)	32,800 (6600)	19,500 (1300)	17,600 (5600)	
**3**	5	n.d.	441 (24)	n.d.	n.d.	n.d.	3450 (320)
	30	10,200 (900)	96 (18)	>100,000	38,400 (6500)	26,200 (4100)	
**4**	5	n.d.	249 (44)	n.d.	n.d.	n.d.	7260 (1350)
	30	4560 (570)	68 (8)	>100,000	21,800 (9700)	7120 (3290)	

^a^ Data shown are mean values ± SEM of 3 separate experiments, each performed in duplicate; “time” represents the preincubation time of enzyme and inhibitor before starting the enzymatic reaction by addition of DMC. ^b^ Data shown are mean values ± SEM of 2 separate experiments, each performed in duplicate. ^c^ Data were previously published [54]. Data were obtained by nonlinear regression of plots v = f([inhibitor]) according to Equation (3) (Section 4.3.2). n.d. denotes “not determined”.

**Table 3:** Summary of key parameters for application of the FA-based assay in cell lysates.

[DMC]/[**R-I-Cad**]	30/0.81 µM
Detection limit	≥0.5 µg/mL hTGase 2
Linear range	0.5–5 µg/mL hTGase 2
[cell lysate]	≤1 g/L (otherwise nonspecific binding of **R-I-Cad** becomes limiting)
Selectivity	Order of enzymatic rate at 30 µM DMC: hTGase 1 > hTGase 2 ≈ hTGase 6 > hFXIIIa > hTGase 3 Control measurements with a selective hTGase 2 inhibitor are recommended.
Further applications	Inhibitor characterization is possible on recombinant TGases and in cell lysates [54].

**Table 4:** Buffer names and their compositions.

Assay buffer A	100 mM MOPS pH 8.0, 3 mM CaCl_2_, 50 µM EDTA
Assay buffer B	100 mM MOPS pH 8.0, 6 mM CaCl_2_, 50 µM EDTA
Enzyme buffer A	100 mM MOPS pH 8.0, 3 mM CaCl_2_, 10 mM TCEP, 20% glycerol (*v/v*)
Enzyme buffer B	100 mM MOPS pH 8.0, 3 mM CaCl_2_, 10 mM DTT, 20% glycerol (*v/v*)
RIPA lysis buffer	150 mM NaCl, 50 mM Tris pH 8.0, 1% NP40, 0.5% SDS (*w*/*v*), 0.5% sodium deoxycholate (*w/v*), 7 µg/mL leupeptin, 1 mM PMSF, 1 mM Na_3_VO_4_, 1 mM DTT, 7 mM NaF
Modified RIPA buffer A	150 mM NaCl, 50 mM Tris pH 8.0, 1 µg/mL leupeptin, 1 mM PMSF, 1 mM Na_3_VO_4_, 1 mM DTT, 5 mM NaF
Modified RIPA buffer B	150 mM NaCl, 50 mM Tris pH 8.0, 7 µg/mL leupeptin, 1 mM PMSF, 1 mM Na_3_VO_4_, 1 mM DTT, 7 mM NaF
5× SDS-PAGE protein loading buffer	312.5 mM Tris pH 6.8, 40% glycerol, 10% SDS, 5% β-mercaptoethanol, 0.1% bromophenol blue
HEPES buffer A	50 mM HEPES pH 8.0, 6 mM CaCl_2_
HEPES buffer B	50 mM HEPES pH 7.4

**Table 5:** Investigated cell lines and appropriate cell culture media (information on origin of cell lines and culture conditions have been obtained from the American Type Culture Collection).

Name	Species	Origin		Medium
A375	human	skin malignant melanoma	primary tumor	DMEM + 10% FCS
MeWo	human	skin malignant melanoma	lymph node metastasis	DMEM + 10% FCS
A2058	human	skin melanoma	lymph node metastasis	DMEM + 10% FCS
MelJuso	human	skin melanoma	primary tumor	RPMI 1640 + 10% FCS
A-431	human	skin epidermoid carcinoma	primary tumor	DMEM + 10% FCS
FaDu	human	pharynx squamous cell carcinoma	primary tumor	DMEM + 10% FCS
NCI-H292	human	lung mucoepidermoid carcinoma	lymph node metastasis	RPMI 1640 + 10% FCS
Hep G2	human	hepatocellular carcinoma	primary tumor	DMEM + 10% FCS
MIA PaCa-2	human	pancreas epithelial cell carcinoma	primary tumor	DMEM + 10% FCS
PanC-1	human	pancreas ductal epithelioid carcinoma	primary tumor	DMEM + 10% FCS
SU.86.86	human	pancreas ductal carcinoma	liver metastasis	RPMI 1640 + 10% FCS
AsPC-1	human	pancreas adenocarcinoma	ascites metastasis	RPMI 1640 + 2 mM glutamine + 1 mM sodium pyruvate + 10% FCS
HPAF-II	human	pancreas adenocarcinoma	ascites fluid	EMEM + 10% FCS
SW1990	human	pancreas adenocarcinoma	spleen metastasis	L15 + 10% FCS
PC-3	human	prostate adenocarcinoma	bone metastasis	F12 + 2 mM glutamine + 10% FCS
MDA-MB-231	human	breast adenocarcinoma	pleural effusion	L15 + 2 mM glutamine + 15% FCS
HT-29	human	colorectal adenocarcinoma	primary tumor	McCoy + 10% FCS
SW480	human	Dukes’ type B, colorectal adenocarcinoma	primary tumor	L15 + 10% FCS
SW620	human	Dukes’ type C, colorectal adenocarcinoma	lymph node metastasis	L15 + 10% FCS
SW403	human	Dukes’ type C, colorectal adenocarcinoma	primary tumor	L15 + 10% FCS
U-87 MG	human	brain glioblastoma	primary tumor	DMEM + 10% FCS
U-251 MG	human	brain glioblastoma astrocytoma	primary tumor	DMEM + 2 mM glutamine + 1% nonessential amino acids + 1 mM sodium pyruvate + 10% FCS
B16-F0	mouse	skin melanoma	primary tumor	DMEM + 10% FCS
B16-F10	mouse	skin melanoma	lung metastasis, 10th generation	DMEM + 10% FCS
MPC	mouse	pheochromocytoma	primary tumor	RPMI 1640 + 10% horse serum + 5% FCS
HPaSteC	human	pancreatic stellate cells		SteCM
HAEC	human	aortic endothelium		ECGM-2
HUVEC	human	umbilical vein endothelium		ECGM-2
HDMEC	human	dermal microvascular endothelium		ECGM-2
hCMEC/D3	human	cerebral microvascular endothelium		ECGM-2

**Table 6:** Antibodies used for Western blot analyses.

**Primary Antibodies**	**Product No.**	**Company**	**Dilution**
anti-TGase 1 polyclonal rabbit	ab103814	Abcam	1:100
anti-TGase 2 monoclonal mouse	ab2386	Abcam	1:500
anti-TGase 3 polyclonal rabbit	ab203229	Abcam	1:500
anti-TGase 6 polyclonal rabbit	ab180959	Abcam	1:1000
anti-factor XIIIa polyclonal rabbit	ab97636	Abcam	1:500
anti-propionyl-CoA carboxylase polyclonal rabbit	ab187686	Abcam	1:5000
anti-β-actin monoclonal mouse	A5316	Sigma-Aldrich	1:1000
**Secondary Antibodies**	**Product No.**	**Company**	**Dilution**
anti-rabbit IgG POD polyclonal goat	A0545	Sigma-Aldrich	1:2000
anti-mouse IgG POD polyclonal rabbit	A9044	Sigma-Aldrich	1:2000/1:10,000

## Data Availability

The data presented in this study are contained within the article.

## Supplementary Materials

The following supporting information can be downloaded at: https://www.mdpi.com/article/10.3390/ijms23094475/s1.
