# Supplementary material for: Application of a Fluorescence Anisotropy-Based Assay to Quantify Transglutaminase 2 Activity in Cell Lysates
Source: Int J Mol Sci. 2022 Apr 19;23(9):4475. doi: 10.3390/ijms23094475 (PMC9104438; doi:10.3390/ijms23094475)
Supplement: Supplementary file 1 [file ijms-23-04475-s001.zip › ijms-1669957-supplementary.pdf]

Supplementary Materials

Application of a Fluorescence Anisotropy-Based Assay to Quantify Transglutaminase 2 Activity in Cell Lysates

Sandra Hauser [1], Paul Sommerfeld [2], Johanna Wodtke [1], Christoph Hauser [2], Paul Schlitterlau [1], Jens Pietzsch [1,3], Reik Löser [1,3,†], Markus Pietsch [2,*,†] and Robert Wodtke [1,*,†]

[1] Helmholtz-Zentrum Dresden-Rossendorf, Institute of Radiopharmaceutical Cancer Research,
Bautzner Landstraße 400, 01328 Dresden, Germany; s.hauser@hzdr.de (S.H.); j.wodtke@hzdr.de (J.W.); paul.schlitterlau@aol.de (P.Sc.); j.pietzsch@hzdr.de (J.P.); r.loeser@hzdr.de (R.L.)

[2] Institute II of Pharmacology, Center of Pharmacology, Faculty of Medicine and University Hospital of
Cologne, University of Cologne, Gleueler Straße 24, 50931 Cologne, Germany; paul.sommerfeld@uk-koeln.de (P.So.); christoph.hauser1@gmx.de (C.H.)

[3] Faculty of Chemistry and Food Chemistry, School of Science, Technische University Dresden,
Mommsenstraße 4, 01069 Dresden, Germany

* Correspondence: markus.pietsch@uk-koeln.de (M.P.); r.wodtke@hzdr.de (R.W.); Tel.: +49-221-478-97737 (M.P.); Tel.: +49-351-260-4033 (R.W.)

† These authors are equally credited as senior authors.

Figure of Contents

Figure S1: **Initial FA values for R-I-Cad, R-S-Cad and F-Cad in the presence of varying DMC concentrations 3**

Figure S2: **Detection of propionyl-CoA carboxylase in cell lysates 4**

Figure S3: **Standard curve for the activity-based ELISA 5**

Figure S4: **Standard curve for the two-site sandwich ELISA 6**

Scheme S1: **Synthesis of inhibitors 3 and 4 ....7**

References **...8**

Figure S1. Initial FA values for R-I-Cad, R-S-Cad and F-Cad in the presence of varying DMC concentrations

Top: Plots of the FA values at start of measurement *versus* DMC concentration in the presence and absence of hTGase 2 and gpTGase 2. The plots clearly show the dramatic increase in the FA values with increasing DMC concentration for **R-I-Cad** and **R-S-Cad**, which is significantly less pronounced for **F-Cad**. The data (mean values ± SEM of 2-4 separate experiments, each performed in duplicate or triplicate) belong to the DMC characterizations toward hTGase 2 and gpTGase 2 which were previously published by us [1, 2], but these plots were not shown. Conditions: pH 8.0, 30°C, 5% DMSO, 500 µM DTT.

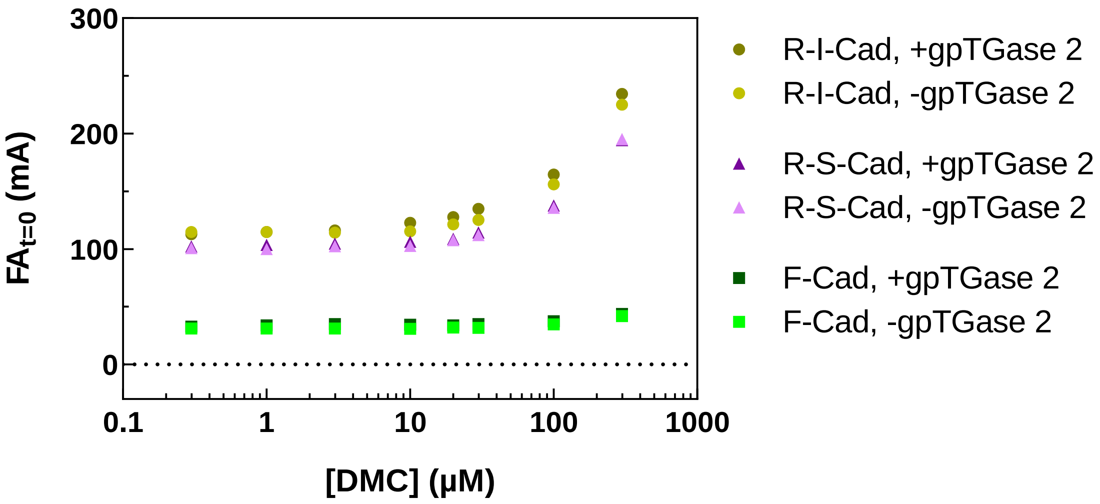

Figure S2. Detection of propionyl-CoA carboxylase in cell lysates

Synthesis of propionyl-CoA carboxylase α subunit (PCCA) in different human cancer cell lines and non-cancer cell line HUVEC. Exemplary western blot lanes for selected cell lines with bands detected at the predicted size of 80 kDa, amongst others. Even though PCCA exhibits a higher molar mass than hTGase 2, it appears rather at 70 kDa, which is in accordance to exemplary data for the used antibody (ab187686, abcam). For loading control, detection of β-Actin was performed. Thermo Scientific™ PageRuler™ Plus Prestained Protein Ladder was acquired with white light illumination and automatically merged to the chemiluminescent image (imager Celvin S).

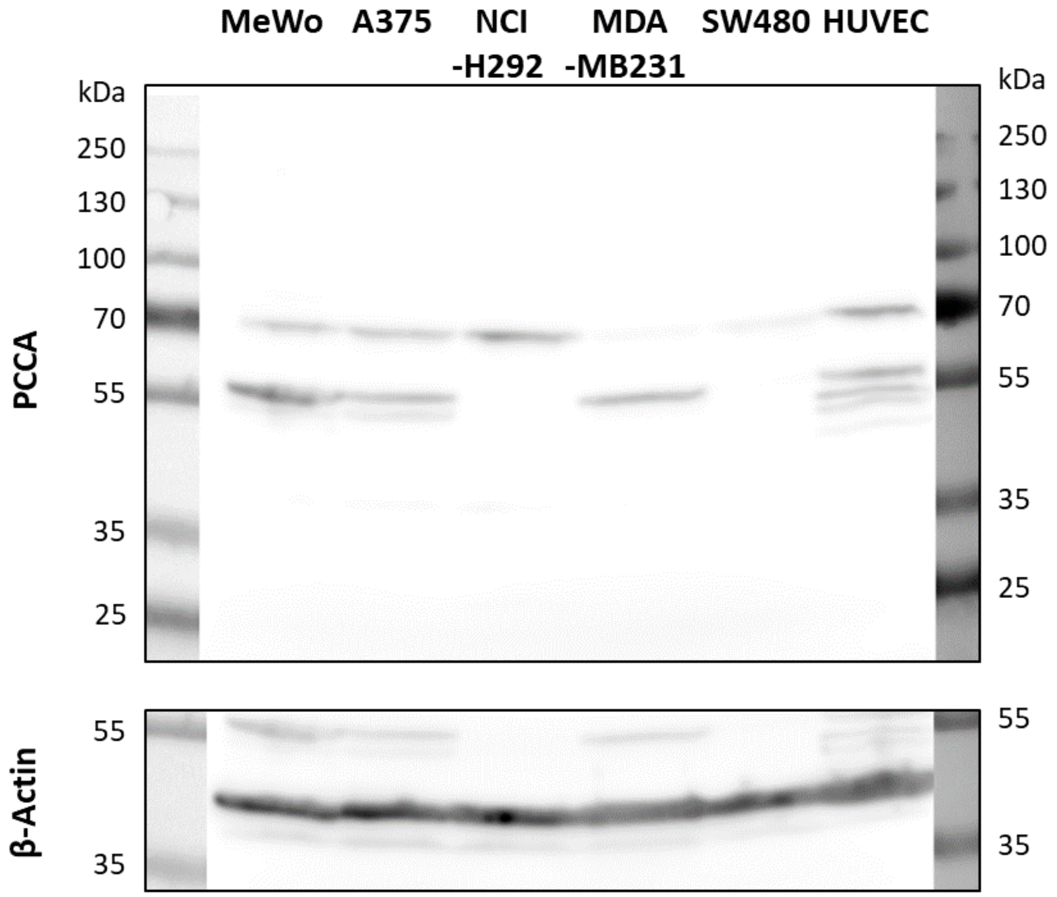

Figure S3. Standard curve for the activity-based ELISA

Plot of relative luminescence units (RLU) as a function of the applied amount of hTGase 2-**4**-complex and non-linear regression using the model of ‘sigmoidal, 4PL, X is log(concentration)’ as implemented in GraphPad Prism (version 9.1.2). Data shown are mean values ± SD of two separate experiments, each performed in triplicate.

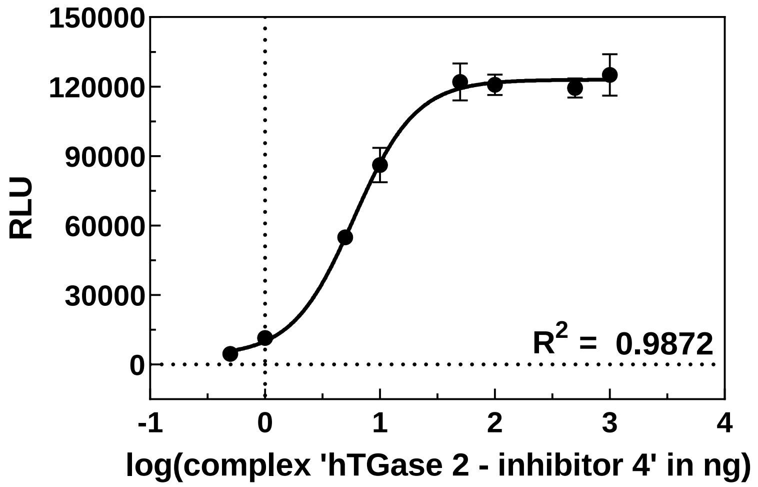

Figure S4. Standard curve for the two-site sandwich ELISA

Plot of absorbance values at 450 nm as a function of the applied concentration of hTGase 2 and non-linear regression using the model of ‘sigmoidal, 4PL, X is log(concentration)’ as implemented in GraphPad Prism (version 9.1.2). Data shown are mean values ± SD of two separate experiments, each performed in duplicate.

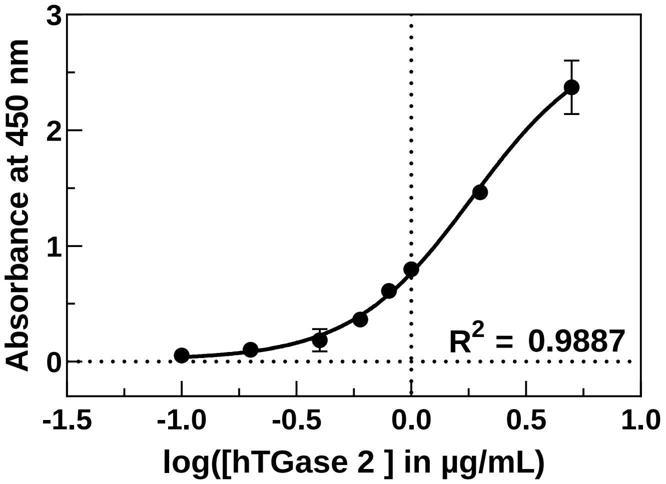

Scheme S1. Synthesis of inhibitors 3 and 4

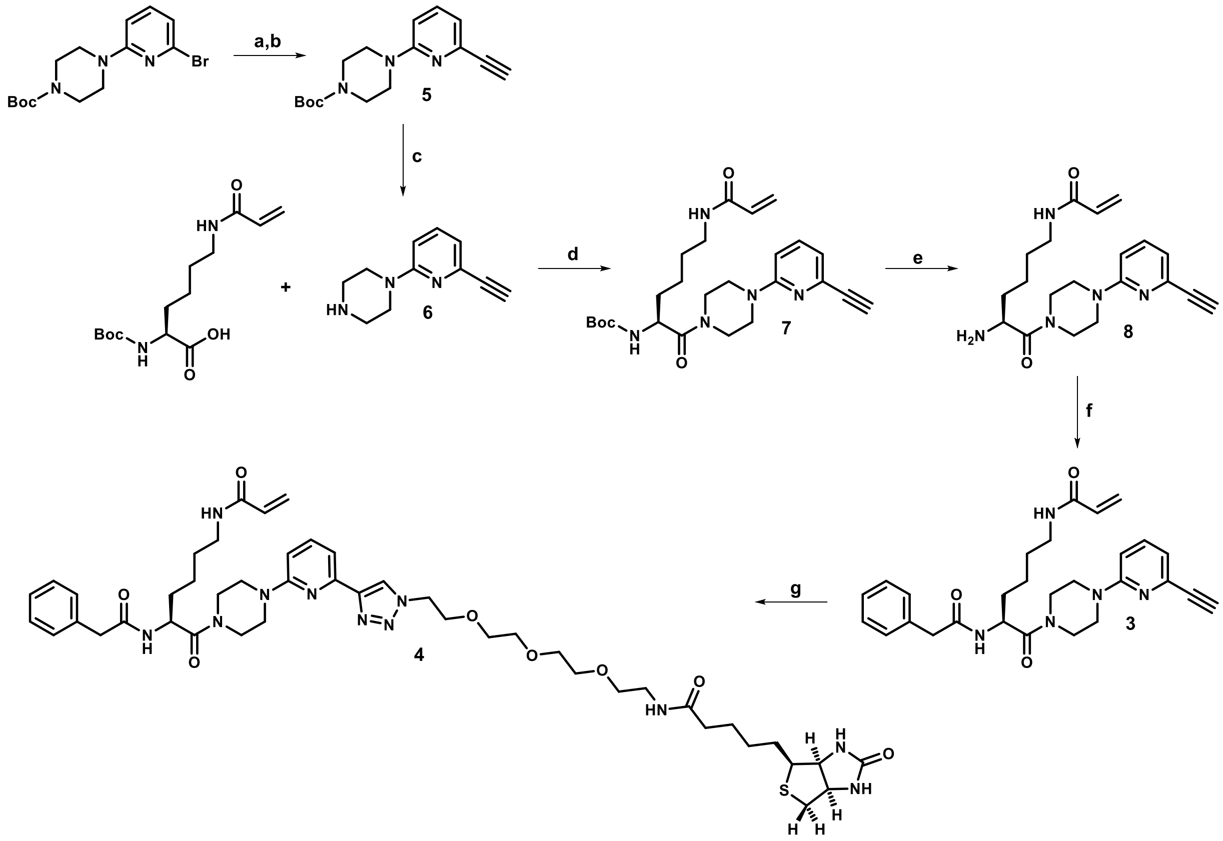

Reagents and conditions: **a)** CuI, $Pd(PPh_3)_4$, trimethylsilylacetylene, diisopropylamine, THF, Ar, 17 h; **b)** TBAF, CH_2Cl_2, 35 min; **c)** TFA/CH_2Cl_2 (1/1), 2 h; **d)** HATU, DIPEA, THF, 1 h; **e)** TFA/CH_2Cl_2 (1/1), 2 h; **f)** phenylacetyl chloride, TEA, CH_2Cl_2, 1 h; **g)** Biotin-PEG3-azide, THPTA, $CuSO_4 \times 5\ H_2O$, sodium ascorbate, *tert*-butanol/water (100:15, v/v), 3 h.

References

1. Wodtke, R.; Hauser, C.; Ruiz-Gomez, G.; Jäckel, E.; Bauer, D.; Lohse, M.; Wong, A.; Pufe, J.; Ludwig, F. A.; Fischer, S.; Hauser, S.; Greif, D.; Pisabarro, M. T.; Pietzsch, J.; Pietsch, M. and Löser, R. N^{ε}-Acryloyllysine piperazides as irreversible inhibitors of transglutaminase 2: synthesis, structure-activity relationships, and pharmacokinetic profiling. *J. Med. Chem.*, **2018**, *61*, 4528-4560.
2. Hauser, C.; Wodtke, R.; Löser, R. and Pietsch, M. A fluorescence anisotropy-based assay for determining the activity of tissue transglutaminase. *Amino Acids*, **2017**, *49*, 567-583.
